# An Analytical Workflow to Quantify Biodegradable Polyesters in Soils and Its Application to Incubation Experiments

**DOI:** 10.1021/acs.est.4c10664

**Published:** 2025-04-18

**Authors:** Mattia Cerri, Flora Wille, Silvan Arn, Thomas D. Bucheli, Franco Widmer, Rhayn Werz, Kristopher McNeill, Alessandro Manfrin, Michael Sander

**Affiliations:** † Institute of Biogeochemistry and Pollutant Dynamics, Department of Environmental Systems Science, Swiss Federal Institute of Technology Zurich (ETH Zurich), 8092 Zurich, Switzerland; ‡ Environmental Analytics, Agroscope, 8046 Zurich, Switzerland; § Molecular Ecology, Agroscope, 8046 Zurich, Switzerland

**Keywords:** Soxhlet extraction, biodegradable polymer, biodegradation, soil, ^1^H-NMR, mulch film

## Abstract

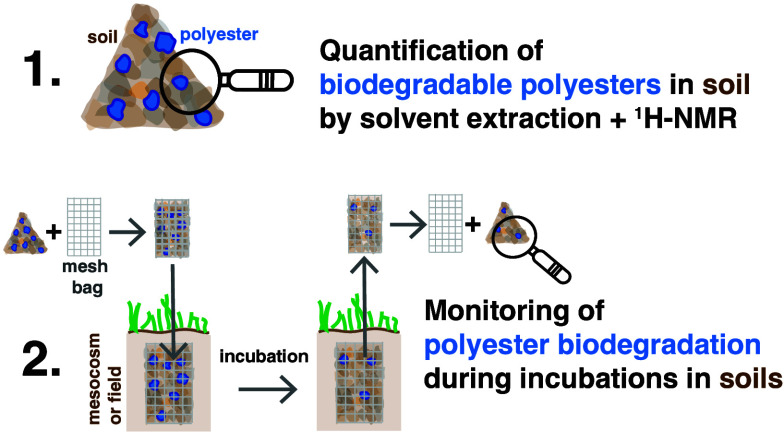

Soil biodegradable polyesters are designed to undergo to microbial utilization in aerobic soils, forming carbon dioxide and microbial biomass. These polyesters are thus viable substitutes for conventional, persistent polymers (e.g., polyethylene) in specific applications for which the transfer of some of the polymers into the soil is inevitable. While polymer biodegradability is often assessed in laboratory incubations using respirometric analysis of formed CO_2_, approaches to accurately quantify biodegradable polyesters in soils and to track their mass loss in field incubations over time remain missing. This study first introduces an analytical workflow combining Soxhlet extraction with proton nuclear magnetic resonance spectroscopy for the accurate, high-throughput, and chemically selective quantification of eight commercially important biodegradable polyesters (i.e., poly(butylene adipate-*co*-terephthalate), polylactic acid, poly(3-hydroxybutyrate-*co*-3-hydroxyhexanoate), poly(3-hydroxybutyrate-*co*-3-hydroxyvalerate), polycaprolactone, polybutylene adipate, polybutylene azelate, and polybutylene succinate), and the nonbiodegradable polymer polystyrene, in six soils spanning a range of types and physicochemical properties. This work introduces an effective sample deployment–retrieval approach that, combined with the analytical method, allows the biodegradation of poly(butylene adipate-*co*-terephthalate) and polylactic acid from a biodegradable mulch film in three agricultural soils to be monitored. In combination, the two parts of this work lay the foundation to accurately quantify and monitor biodegradable polymers in soils.

## Abstract 2

This work presents an analytical workflow to quantify biodegradable polyesters in soils and, combined with a mesh-bag sample deployment−retrieval approach, to monitor polyester biodegradation in soils.

## Introduction

Biodegradable polymers are increasingly recognized as being fundamental to achieving a circular plastic economy and to addressing environmental plastic pollution.^1,2^ While, for many applications, plastic reuse and recycling are preferable options, as they retain at least a minimum of value in the use chain of the polymers,^1^ the use of biodegradable polymers is particularly beneficial in applications in which plastic products are directly employed in the open environment but cannot be collected in their entirety after use, and/or for which the collected fractions are too damaged or soiled to be reused or recycled.^3,4^ Prime examples of these applications include agricultural plastics^5−11^ (e.g., thin mulch films, plant fixing clips, control-release carriers for agrochemicals, seed coatings) and tree shelters,^12−14^ all of which have soils as the anticipated receiving environment.

Most commercially relevant biodegradable plastics are currently manufactured from polyesters,^15−17^ including poly(butylene adipate-*co*-terephthalate) (PBAT), polylactic acid (PLA), poly(butylene succinate) (PBS), and polyhydroxyalkanoates. During the biodegradation process in soils, these polyesters undergo microbial metabolic utilization, resulting in conversion of their carbon to CO_2_ (and CH_4_, under anaerobic conditions) and microbial biomass. The safe environmental application of biodegradable plastics requires that this conversion reproducibly reaches adequate end points within defined times. Exactly for this purpose, biodegradability testing and certification standards for different receiving environments have been developed.^18−20^ In the case of soils, certification standards rely on quantifying the conversion of the polymer carbon to CO_2_ during aerobic soil incubations in the laboratory;^19,21−23^ for example, the European Norm EN 17033 for soil biodegradable mulch films requires that at least 90% of the polymer carbon (either absolute or relative to a biodegradable reference material, e.g., cellulose) is converted to CO_2_ within two years of laboratory soil incubation under constant, controlled conditions.^21^

Laboratory incubations are well-suited to assess polymer biodegradability under controlled, reproducible conditions with high precision. However, these benefits come at the cost of representativeness, since laboratory incubations are only a reductionist miniaturization of the actual polymer receiving environment (e.g., agricultural soils) and do not capture all the highly variable environmental conditions that do affect biodegradation (e.g., seasonal temperature changes and wet–dry cycles). Instead, laboratory soil incubations are commonly run under conditions that favor microbial activity (e.g., constant temperatures between 20 and 28 °C and favorable soil water contents^21^) and, therefore, likely overestimate biodegradation rates compared to *in situ* conditions.^19,24^

Assessing polymer biodegradation directly in soils outside of the laboratory has, however, proven challenging, as the respirometric analysis of polymer-derived CO_2_ cannot readily be implemented in larger mesocosms nor in the field.^25,26^ Previous field studies, therefore, have often relied on indirect and, at best, semiquantitative methods to monitor biodegradation (e.g., polymer mass loss over time based on gravimetric analysis of fragments^27−30^). To overcome this deficit, analytical methods that allow the accurate quantification of residual biodegradable polymers in soils are needed.

Proton nuclear magnetic resonance spectroscopy (^1^H-NMR) has been shown to be a viable method to quantify polymers with different chemistries, after their appropriate extraction from the environmental matrix.^31−36^ We recently introduced an analytical method^37^ coupling ^1^H-NMR to Soxhlet extraction with the potential for routine quantification of polyesters in soils. Solvent extraction has the unique benefit that micro- and nano-meter sized polymer particles, if present, are dissolved into individual polymer chains and then extracted–thereby ensuring that polymers within these particle size fractions are also quantified. The original method was validated for PBAT, and also shown to enable the quantification of both PBAT and PLA components of one commercial biodegradable mulch film in a single soil.^37^ Yet, it remains to be demonstrated that the principle of this analytical approach is broadly applicable to commercially relevant biodegradable polyesters, across different soils, with high accuracy, sensitivity, and high sample throughput. The latter is critical for field incubations, which typically investigate multiple contrasts (e.g., multiple time points for multiple soils) and, thus, can require relatively large sample numbers. Furthermore, the analytical method needs to be complemented with an approach to deploy samples in soil in the field and, after incubation, retrieve any residual polymers in their entirety for the analysis.

The goal of this work is twofold. First, this work aims at advancing a universally applicable analytical workflow for the accurate and high-throughput quantification of major biodegradable polyesters across agricultural soils. Second, in a proof-of-concept, this work aims at monitoring the biodegradation of selected polyesters during mesocosm soil incubations (as a proxy for field incubations), by introducing and validating a sample deployment–retrieval approach based on mesh bags and combining it with the analytical workflow developed in the first part.

The methodological work in the first part relies on spike–recovery experiments. Two alternative procedures are presented to address the potential interference from soil organic matter (SOM) co-extracted during the Soxhlet extraction^37^ on polyester quantification: a methanol (MeOH) pre-extraction step, to selectively remove SOM from the samples before polyester extraction, and matrix-matching with soil-only samples, to correct the ^1^H-NMR spectra at the data processing stage. The limits of detection (LOD) and quantification (LOQ) for PBAT and PLA (from one of the mulch films) are established for both options and in two soils. The effect of shorter extraction times (down to 30–60 min extraction compared to 480 min, as used previously^37^) on polyester recoveries is assessed. In addition, the broad applicability of the method is demonstrated with a total of eight biodegradable polyesters (PBAT, PLA, poly(3-hydroxybutyrate-*co*-3-hydroxyhexanoate) (PHBH), poly(3-hydroxybutyrate-*co*-3-hydroxyvalerate) (PHBV), polycaprolactone (PCL), polybutylene adipate (PBA), polybutylene azelate (PBAz), and polybutylene succinate (PBS)) and one nonbiodegradable polymer (polystyrene, PS). In the second part, a sample deployment–retrieval approach based on polypropylene (PP) mesh bags is presented. Such bags are then used to incubate one biodegradable mulch film (containing PBAT and PLA) and PHBH (as positive control) in soil mesocosms in three different soils to monitor polymer biodegradation.

## Materials and Methods

### Soils

Three standard soils (i.e., LUFA 2.1, 2.4 and 6S) sieved to 2 mm were acquired from LUFA Speyer (Germany; May 2018) and stored as received at 4 °C in the dark until use. Three additional soils were included, one collected from a noncultivated ecological buffer strip^38^ (hereafter named AGR-1) and two from agricultural fields (hereafter, AGR-2 and AGR-3) in November 2020 at Agroscope (Reckenholz, Switzerland). The AGR soils were also sieved to 2 mm and stored at 4 °C in the dark until use. Table 1 summarizes the texture and main physicochemical properties of the soils. Further information on the AGR soils, including the sampling procedure, is provided in Section 1 of the Supporting Information (SI).

**Table 1:** Key Physicochemical Properties of the Six Soils Included in This Study

	(g/g_dry soil_ (%))						
soil	sand	silt	clay	texture^a^	pH	TOC^b^ (g_C_/g_dry soil_ (%))	TN^c^ (g_N_/g_dry soil_ (%))
AGR-1	30.5	49.0	20.5	loam	4.9	1.52	0.19
AGR-2	12.1	52.0	35.9	silty clay loam	7.0	3.40	0.41
AGR-3	22.7	44.1	33.2	clay loam	6.9	2.02	0.25
LUFA 2.1	86.1	10.2	3.7	loamy sand	4.9	0.71	0.06
LUFA 2.4	32.1	41.6	26.3	loam	7.3	2.03	0.22
LUFA 6S	23.8	35.3	40.9	clay	7.2	1.77	0.18

a Soil texture according to the United States Department of Agriculture (USDA) classification system (sand, particle diameters of 0.05–2.0 mm; silt, diameters of 0.002–0.05 mm; clay, diameters of <0.002 mm).^39^

b Total organic carbon content expressed as the percentage of carbon (C) by mass of the total soil dry mass.

c Total nitrogen content expressed as the percentage of nitrogen (N) by mass of the total soil dry mass.

### Polymers and Mulch Films

The following polymers and commercial mulch films were included in this study: PHBH (Aonilex X151C, Kaneka), PHBV (403105, Sigma-Aldrich), PCL (764105, Sigma-Aldrich), PBA, PBAz, PBS (all three provided by BASF SE Germany), PS (ST316090/3, Goodfellow), and the biodegradable mulch films Bio Mulchfolie 32.00009 (hereafter named MF-R, gvz-rossat SA, Switzerland), Biofolie 15 μ (MF-S, Sansonnens FG Frères SA, Switzerland), and ecovio M2351 (MF-E, BASF SE, Germany). All mulch films were 15 μm thick and composed of PBAT and PLA at different mass percentages (MF-R: 56 ± 1% PBAT and 14 ± 1% PLA; MF-S: 70 ± 1% PBAT and 4 ± 1% PLA; MF-E: 67 ± 1% PBAT and 7 ± 1% PLA; average ± standard deviation, n = 45). All polymer chemical structures, polymer purities, and the monomer ratios of PHBV, PHBH, and PBAT in the mulch films are provided in Section 2, SI.

### Spike–Recovery Experiments

For spike–recovery experiments, 20 g of freeze-dried soil (frozen at −20 °C overnight, and then freeze-dried for 48 h, Alpha 2–4 LD plus, Christ, Germany, at 0.01 mbar) were transferred to cellulose extraction thimbles. Known amounts of a polymer (20 mg) or mulch film (35 mm diameter discs, average mass ∼17 mg) were then manually mixed into the soil, extracted and quantified as described below.

### Polymer Extraction

Extractions were carried out on freeze-dried soil samples in cellulose extraction thimbles (VWR, 516–0252P). Each thimble was capped with a small wad of glass wool (Merck, 1.04086) and placed in a Soxhlet extractor chamber (heating apparatus: R306S, behr Labor-Technik; extractor chamber: EZ30H, behr Labor-Technik, 30 mL volume). The extraction procedure consisted of either a single CHCl_3_:MeOH (9:1 v/v) extraction step (K977, VWR and 1.02444, Merck, respectively, both HPLC grade) or two sequential steps: a MeOH pre-extraction step to remove extractable SOM, followed by the CHCl_3_:MeOH extraction (9:1 v/v) step to extract the polymer(s). MeOH pre-extractions were conducted at 100% heating power (360 W) under continuous reflux for 30 min in 100 mL clean round-bottom flask containing 70 mL MeOH. Polymer extractions were conducted at 90% heating power under continuous reflux with clean 100 mL round-bottom flasks containing 70 mL of a 9:1 v/v CHCl_3_:MeOH mixture, for a total of 60 min (corresponding to 18 to 24 extraction cycles), unless specified differently. Teflon stir bars were added to the flasks to avoid flash boiling. After the polymer extraction, the CHCl_3_:MeOH was evaporated off. Each flask was connected to a vacuum line for 20 min to ensure complete solvent removal. The dried extracts were then reconstituted in 3 mL deuterated chloroform (CDCl_3_) (DLM-7–100S, CIL) containing the internal standard (IS) 1,4-dimethoxybenzene (DMB; used for all polyesters) (D0629, Tokyo Chemical Industry) or 1,4-bis(trifluoromethyl)benzene (TFB, B1408, Tokyo Chemical Industry; used for PS because of overlapping peaks in the ^1^H-NMR spectra of PS and DMB) at a known concentration (∼1.5 mg/mL) and sonicated for 20 s at 25 °C. Selected extracts from the MeOH pre-extractions were reconstituted in the same way to test for potential (undesired) polymer extraction in this step.

### Polymer Quantification

Concentration standards were prepared by dissolving known amounts of a given polymer or mulch film in CDCl_3_ containing the IS. Similarly, for all spike–recovery experiments (first part of the work) and for the incubation experiments (second part), dried polymer extracts were reconstituted in CDCl_3_ containing the IS specified above. The acquisition parameters for the ^1^H-NMR routine are reported in Section 3, SI. Quantification of polymers by ^1^H-NMR spectroscopy is described in detail in the SI and previous work.^37^ Briefly, the mass of each polymer in a given sample was calculated according to^40^
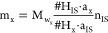
1where m_*x*_ (g) is the mass of the polymer x, M_w_*x*__ (g/mol) is the molecular weight of the polymer repeat unit, a_*x*_ and a_IS_ (arbitrary units) are the areas of the proton peaks in the ^1^H-NMR spectra at the characteristic chemical shifts chosen for quantification of the polymer x and IS, respectively, #H_IS_ and #H_*x*_ are the number of protons per single molecule/repeat unit contributing to the signal of these peaks, and n_IS_ is the known amount (mol) of IS in a given sample. The linearity, accuracy and unbiasedness of the ^1^H-NMR response underlying eq 1 have been previously established.^37^

^1^H-NMR spectra were processed in MestReNova 14.2.0. The spectra were first referenced to the ^1^H peak of residual nondeuterated CHCl_3_ in CDCl_3_ (at chemical shift δ = 7.26 ppm^41^). The phase was manually corrected, and the baseline set to an intensity of approximately zero with a piecewise linear correction. All characteristic peaks were then manually integrated using MestReNova integration routine. Annotated ^1^H-NMR spectra, the characteristic peaks chosen for quantification, and values of M_w_*x*__, #H_*x*_, and #H_IS_ of each polymer are provided in Section 3, SI.

All further data analysis was performed in R 4.3.2^42^ with the IDE RStudio 2023.06.1^43^ (see Section 4, SI, for a complete list of packages). The results of the extractions are provided as average and standard deviation of the percentage of the mass of polymer added to the respective samples.

### Limits of Detection and Quantification

The LOD and LOQ for PBAT and PLA in MF-R were determined in soils AGR-2 and LUFA 6S using a linear calibration method^44^ with six concentration values (0 to 0.75 mg polymer/mL). These soils were selected because the co-extracted SOM interfered with polyester quantification due to overlaps in different regions of the ^1^H-NMR spectrum (see Section 5, SI). The LOD and LOQ were determined for four different cases: MF-R dissolved in pure CDCl_3_ (as best case scenario, with no matrix interference); MF-R dissolved in the CHCl_3_:MeOH extracts of either AGR-2 or LUFA 6S (extraction time: 60 min); MF-R dissolved in the CHCl_3_:MeOH extracts of either AGR-2 or LUFA 6S (extraction time: 60 min) after each soil had been pre-extracted with MeOH to remove extractable SOM (extraction time: 30 min); and MF-R dissolved in the CHCl_3_:MeOH extracts of either AGR-2 or LUFA 6S (extraction time: 60 min) and subsequently matrix-matched (i.e., spectra of samples containing only the corresponding soil were subtracted from the spectra of the samples containing both the polymer and the soil to correct for the SOM background). All samples were prepared as duplicates.

Linear calibration curves (^1^H-NMR signal intensity as a function of the polymer concentration) were constructed for each of the four cases. The LOD was determined using eq 2 for pure CDCl_3_ (i.e., no matrix interference):
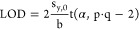
2and eq 3 for all other cases (for which the matrix interference of the soils needs to be accounted for^44^):
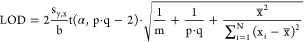
3where b is the slope of the calibration curve, s_y,0_ is the standard deviation of the peak areas of the blank samples (i.e., 0 mg/mL polymer), t(α, p·q–2) ∼ 1.81 is the value of the Student-t distribution for a type I error probability α = 0.05 (one-sided test) and p·q degrees of freedom, p = 6 is the number of different concentration standards included in the calibration curve, q = 2 is the number of replicates of each concentration standard, s_y,x_ is the standard deviation of the residuals of the calibration curve, m = 1 is the number of repeated measurements of each calibration standard, x_i_ is the concentration of the i-th calibration standard, N = p·q is the total number of calibration standards, and x̅ is their average concentration. The factor 2 in front of eq 3 is included to account for a symmetric type I and type II error probability. Examples of the calibration curves are shown in Section 5, SI.

The LOQ was then calculated according to eq 4:^44^
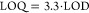
4

As PBAT is a copolymer, the LOD and LOQ were determined for both its 1,4-butanediol-adipic acid (BA) and 1,4-butanediol-terephthalic acid (BT) repeat units.

### Polyester Incubations to Monitor Biodegradation in Soil over Time

Known amounts of PHBH powder and MF-R film (containing both PBAT and PLA) were incubated in three soil mesocosms constructed from PP boxes (60 cm × 40 cm × 32.3 cm L × W × H; Rako Utz) filled with 70 kg of thoroughly mixed soil (one box per each AGR-1, AGR-2, or AGR-3). Each mesocosm was lined with a PP fleece (sandpit fleece, 06059, Windhager) and had holes drilled in the bottom of the box. The holes were strung with capillary PP fleece wicks to ensure efficient water drainage. The mesocosms were placed on tables in a heated greenhouse (minimum, median and maximum temperature during the entire incubation period: 13 °C, 18 °C, and 34 °C), regularly irrigated with stored rainwater using a custom-made system (average artificial irrigation rate during the entire incubation: 2.3 mm/d) and received artificial lighting (in addition to natural light) daily from 6 to 10 pm. The samples were prepared by adding 20 g of sieved soil and either PHBH (20 mg of powder) or MF-R discs (35 mm diameter, average mass ∼17 mg) into small (∼4 cm × 9 cm) PP mesh bags (PROPYLTEX 05–150/34, Sefar S, mesh size 150 μm, closed by heat-sealing). Polypropylene was chosen as it does not dissolve in either MeOH or CHCl_3,_ used in the later extraction steps. The bags were carefully filled to ensure that the PHBH powder or mulch film discs were completely enclosed by soil, then buried at approximately 20 cm of depth in the mesocosm filled with the respective soil. English ryegrass *Lolium perenne* “Arvicola”^45^ was sown to cover each mesocosm.

A first set of samples was retrieved after four months of incubation and served to optimize the sample preparation procedure for the subsequent extraction and quantification of residual polyesters. A second set of samples was retrieved after six months, worked up using the optimized sample preparation procedure established with the four-month samples, then extracted (30 min MeOH pre-extraction; 60 min CHCl_3_:MeOH polymer extraction) to quantify the residual amounts of PHBH, and of PBAT and PLA from MF-R.

## Results and Discussion

### Analytical Workflow to Quantify Biodegradable Polyesters in Soils

The first part of this work presents method developments to advance an analytical workflow for the accurate and sensitive quantification of a broad set of polyesters in diverse soils at high sample throughput.

#### Addressing Interference from Co-extracted SOM

Soxhlet extracts of soil samples can contain SOM that interferes with polymer quantification, due to overlaps in the ^1^H-NMR spectra of extracted SOM constituents and polymers.^37^ Indeed, initial spike–recovery experiments of MF-R in soil AGR-2 (the soil with the highest organic carbon content; Table 1) showed overquantification of both PBAT and PLA in the MF-R (i.e., PBAT and PLA recoveries of 108 ± 1% and 123 ± 8%, respectively, average ± standard deviation, n = 3). To address this bias, a pre-extraction with a solvent that selectively removes SOM, but not the polymers, was tested. Despite the relatively high water-solubility of SOM, a water extraction was excluded, as it may introduce polyester losses due to the hydrolysis of ester bonds.^46,47^ Instead, MeOH was chosen for pre-extraction (30 min), given that all polyesters tested herein have limited solubility in this solvent, and that it is used as cosolvent in the subsequent polymer extraction step (note that MeOH serves to increase extraction efficiency by competitively suppressing H-bonding between H-bond donors on soil particle surfaces and the H-bond accepting ester bonds in the polyesters^37^).

The MeOH pre-extraction effectively removed extractable SOM components, thereby increasing the signal-to-noise ratio in the ^1^H-NMR spectra of PBAT and PLA extracted in the subsequent (second) CHCl_3_:MeOH extraction step (Section 5, SI). More importantly, the MeOH pre-extraction eliminated overquantification of PBAT and PLA (i.e., recoveries of 98 ± 1% and 101 ± 2%, respectively, average ± standard deviation, n = 3). Finally, reconstitution of the MeOH pre-extracts into CDCl_3_ showed that neither PBAT nor PLA was extracted in this step. The MeOH pre-extraction step was thus included routinely in all subsequent experiments, unless specified differently.

#### Establishing Short Extraction Times to Ensure High Sample Throughput

The time needed for complete polyester extraction depends on several factors, including the specific polyester–soil combination, the degree of compaction of the soil sample (as this affects solvent transfer into the sample; see below), the mass loading in the extraction thimble, as well as the number of extraction cycles per unit time supported by the apparatus. Using a series of spike–recovery experiments with MF-R in soil AGR-2, the viability of short polymer extraction times was tested. Sets of triplicate samples were pre-extracted for 30 min with MeOH, then extracted with CHCl_3_:MeOH for varying times from 10 to 240 min. As shown in Section 6, SI, extraction times as short as 30 to 60 min were found to be sufficient for the complete extraction of both PBAT and PLA (and for most other tested polyesters, as shown in the next section and the results in Table 2).

**Table 2:** Recoveries of Poly(butylene adipate-*co*-terephthalate) (PBAT) and Polylactic Acid (PLA) from the Three Biodegradable Mulch Films MF-R, MF-S, and MF-E and of the Polymers Poly(3-hydroxybutyrate-*co*-3-hydroxyhexanoate) (PHBH), Poly(3-hydroxybutyrate-*co*-3-hydroxyvalerate) (PHBV), Polycaprolactone (PCL), Polybutylene Adipate (PBA), Polybutylene Azelate (PBAz), Polybutylene Succinate (PBS), and Polystyrene (PS) Added to Six Soils and Extracted in CHCl_3_:MeOH (9:1 v:v, 30 min), after a MeOH Pre-extraction (30 min) to Remove Extractable Soil Organic Matter^a^

polymer		soil (extraction recoveries of polymer (% of added mass, average ± sd, *n* = 3))					
		AGR-1	AGR-2	AGR-3	LUFA 6S	LUFA 2.1	LUFA 2.4
MF-R	PBAT	101 ± 2	98 ± 1	99 ± 1	98 ± 1	99 ± 1	98 ± 1
	PLA	102 ± 1	101 ± 2	99 ± 1	97 ± 1	99 ± 1	97 ± 1
MF-S	PBAT	102 ± 1	97 ± 3	100 ± 2	92 ± 1	96 ± 1	91 ± 1
	PLA	106 ± 3	100 ± 3	103 ± 3	95 ± 2	95 ± 1	95 ± 1
MF-E	PBAT	99 ± 1	98 ± 2	97 ± 1	94 ± 2	96 ± 1	95 ± 1
	PLA	102 ± 1	98 ± 3	100 ± 3	93 ± 3	94 ± 1	94 ± 2
PHBH		99 ± 2	99 ± 1	101 ± 1	99 ± 4	100 ± 2	95 ± 5
PHBV		nd^c^	100 ± 3	nd^c^	101 ± 4	nd^c^	nd^c^
PBA		nd^c^	96 ± 1 (∼4%)^b^	nd^c^	95 ± 2 (∼3%)^b^	nd^c^	nd^c^
PBS		nd^c^	91 ± 2	nd^c^	89 ± 5	nd^c^	nd^c^
PBAz		nd^c^	93 ± 1 (∼7%)^b^	nd^c^	92 ± 1 (∼3%)^b^	nd^c^	nd^c^
PCL		nd^c^	92 ± 1 (∼8%)^b^	nd^c^	93 ± 1 (∼9%)^b^	nd^c^	nd^c^
PS		nd^c^	100 ± 4	nd^c^	100 ± 2	nd^c^	nd^c^

a All values are expressed as a percentage of the mass of the polymer initially added to the samples.

b Percentage of the total mass of the polymers detected in the reconstituted MeOH pre-extraction (30 min) of soil organic matter (triplicate samples were pooled for analysis).

c Not determined.

#### Demonstrating Complete Recoveries for a Broad Set of Polymer–Soil Combinations

The broad applicability of the polyester extraction and quantification workflow was demonstrated with spike–recovery experiments with a total of six biodegradable polyesters, three commercial biodegradable mulch films (i.e., MF-R, MF-S, and MF-E, each containing PBAT and PLA), and PS, in a total of six agricultural soils. In all cases, samples were extracted for 30 min with MeOH to remove extractable SOM, then for 30 min with CHCl_3_:MeOH to recover the polymers. PBAT and PLA (from biodegradable mulch films) and PHBH were extracted from all soils, since biodegradable mulch films are the focus of this work, while PHBH may be used as positive control in incubation studies. The remaining polymers were extracted from soils AGR-2 and LUFA 6S, which were selected since their co-extracted SOM causes interference with polymer quantification in different regions of the ^1^H-NMR spectra (see also Section 5, SI).

Complete recoveries (i.e., 97–100% of the polymer mass initially added to the soils, see Table 2) were obtained for PHBH and PHBV from all tested soils, for the PBAT and PLA components of MF-R, MF-S, for MF-E from soils AGR-1, AGR-2 and AGR-3, and for the PBAT and PLA components of MF-R from soils LUFA 2.1, LUFA 2.4 and LUFA 6S. Complete recoveries were also obtained for PS from all tested soils (Table 2).

Recoveries of PBAT and PLA from MF-S and MF-E from soils LUFA 2.1, LUFA 2.4, and LUFA 6S, for PBS in soils AGR-2 and LUFA 6S, and for PBA, PBAz, and PCL were slightly incomplete (i.e., 89–96% of the spiked polymer masses) (Table 2). In the cases of PBA, PBAz, and PCL, the extracts from the MeOH pre-extractions were found to contain small amounts of the respective polyesters (i.e., 3–9% of the initial polymer mass; Table 2). Adding the amounts in the MeOH pre-extracts to those in the CHCl_3_:MeOH extracts yielded closed mass balances. This finding highlights the importance of carefully testing for potential polymer losses during the MeOH pre-extraction in future studies employing this approach. In all other cases, the MeOH pre-extracts did not contain any detectable amount of the respective polymer. However, reproducible recoveries above 80% are commonly considered acceptable for analytical methods,^48−51^ and, in such cases, the bias introduced by an incomplete extraction can be accounted for with recovery factors.^49^ Note that mesocosm incubations (see below) were conducted with polymer–soil combinations that showed complete recoveries and, therefore, did not require the use of recovery factors. Overall, the high and, in most cases, complete recoveries highlight that the analytical method is broadly applicable to a large set of polymer–soil combinations. In future studies, similar spike–recovery experiments need to be carried out to confirm complete recoveries for the polymer–soil combination(s) of interest.

#### Determination of the Limit of Detection and Limit of Quantification for Two Selected Polyesters

The LOD and LOQ depend on the peaks in the ^1^H-NMR spectrum chosen for quantification, the amount and composition of co-extracted SOM, and the number of measurement scans in the ^1^H-NMR routine (Section 5, SI). Copolymers, such as PBAT, PHBH, and PHBV, have distinct LOD and LOQ values for each of their repeat units.

Herein, the LOD and LOQ for PBAT (both for the BA and BT units) and PLA based on calibration standards are reported for different matrices. As expected, the LOD and LOQ were lowest (i.e., 0.3 and 1 μg/mL, respectively) for concentration standards prepared directly in pure CDCl_3_ (i.e., in the absence of interfering SOM; Table 3). Conversely, the LOD and LOQ were highest (i.e., 26–72 and 86–263 μg/mL, respectively; approximately 70 to 240-fold higher than in pure CDCl_3_) for concentration standards prepared in the CHCl_3_:MeOH extracts of AGR-2 and LUFA 6S, without MeOH pre-extraction or correction by matrix-matching. By comparison, the LOD and LOQ were approximately 25% lower (on average) for concentration standards prepared in the CHCl_3_:MeOH extracts of soils AGR-2 and LUFA 6S after their pre-extraction with MeOH, and approximately 45% lower (on average) for the matrix-matched standards. Therefore, both MeOH pre-extraction and matrix matching are viable procedures to improve the LOD and LOQ of the analytical method. The choice of which procedure to implement, if needed, should weigh the positive and negative aspects of both. For example, in terms of sample processing, MeOH pre-extraction and matrix-matching require comparable effort and time (i.e., sequential extractions versus extractions of polymer-free soil samples). Yet, matrix-matching requires additional time for ^1^H-NMR analysis and data processing. Furthermore, for matrix-matching, dedicated polymer-free samples need to be included in the experimental design in advance (for instance, for each time polymer samples are retrieved during an incubation). Conversely, for MeOH pre-extraction, selected MeOH extracts need to be worked up to demonstrate that no polymer is lost in this step.

**Table 3:** Limits of Detection (LOD) and Quantification (LOQ) of the Poly(butylene adipate-*co*-terephthalate) (PBAT) and Polylactic Acid (PLA) Components of Biodegradable Mulch Film MF-R in Soils AGR-2 and LUFA 6S^a^

		LOD/LOQ μg repeat unit/mL (μg of repeat unit/g of soil^c^)						
		CDCl_3_ (pure)	AGR-2 soil extract			LUFA 6S soil extract		
			No SOM^b^ removal, no correction	SOM removal by MeOH pre-extraction	Correction by matrix matching	No SOM removal, no correction	SOM removal by MeOH pre-extraction	Correction by matrix matching
PBAT	BT	0.3/1	30/97	7/25	19/65	59/199	53/178	12/30
			(4/15)	(1/4)	(3/10)	(9/30)	(7/27)	(1/6)
	BA	0.3/1	26/86	22/72	9/30	55/180	46/152	48/159
			(4/13)	(3/11)	(1/4)	(8/27)	(7/23)	(7/24)
PLA		0.3/1	43/143	37/121	23/76	72/236	54/178	48/158
			(7/22)	(5/18)	(3/11)	(11/35)	(8/27)	(7/24)

a Since PBAT is a random copolymer, the LOD and LOQ are reported for both its 1,4-butanediol–adipic acid (BA) unit and 1,4-butanediol–terephthalic acid (BT) unit (see also Section 3, SI).

b SOM, soil organic matter.

c In this study, 20 g of soil was extracted and reconstituted in a final sample volume of 3 mL of CDCl_3_. The LOD and LOQ are dependent on the ratio of soil extracted to the volume of solvent used to reconstitute the extract.

### Soil Mesocosm Incubations

The second part of this work introduces an approach to deploy polymer samples in soils and retrieve them after incubation to monitor their biodegradation over time. The approach relies on sealing the polymer(s) together with homogenized and sieved soil into PP mesh bags and is demonstrated herein with soil mesocosm incubations in a greenhouse as a proxy for field incubations.

#### Effect of Sample Preparation on Extraction Efficiencies

To ensure that incubated samples could be adequately prepared for polymer extraction, a set of polymer samples (i.e., mesh bags filled with 20 g AGR-2 and a disc of MF-R) was incubated in soil mesocosm and retrieved after 4 months. Freeze-drying of these samples resulted in highly compacted soil blocks inside the mesh bags. To determine if this compaction affected the extraction of residual PBAT and PLA, the samples were divided into three groups: two groups to test different pretreatments (i.e., coarse crushing of the soil block inside the mesh bag or removal of the soil block from the mesh bag and manual grinding on glassine paper) and one group as control (i.e., the soil block was left in the mesh bag with no further treatment), before being transferred to extraction thimbles. All samples, including the mesh bags, were then pre-extracted to remove SOM (30 min, MeOH), and sequentially extracted twice (30 min each, CHCl_**3**_:MeOH) to assess whether all residual polymer was extracted in the first extraction step. Note that the total masses of PBAT and PLA extracted from these samples do not add up to 100% of their initial value if the polyesters have biodegraded over four months of incubation.

**Figure 1:**
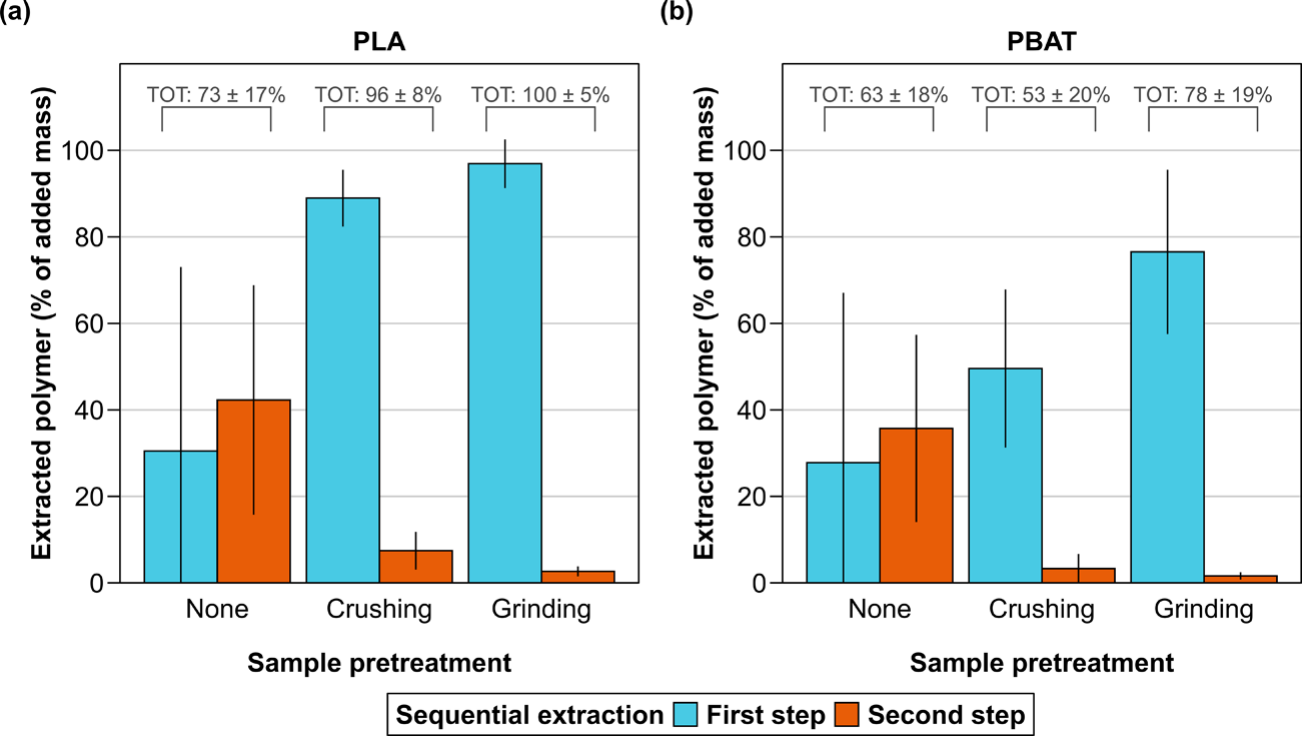
Effect of sample pretreatment on the masses of (a) polylactic acid (PLA) and (b) poly(butylene adipate-*co*-terephthalate) (PBAT) from mulch film MF-R extracted from soil AGR-2 after four months of incubation in greenhouse soil mesocosms. The samples were incubated inside polypropylene mesh bags. The masses are expressed as a percentage of the polymer masses added to the mesh bags before the incubation. The heights of the bars represent the average of each group, and the error bars the corresponding standard deviation (*n* = 3). The values above the brackets (labeled TOT for “total”) correspond to the sum and standard deviation of the sum (*n* = 3) of the masses of each of the two polymers extracted over the two sequential extraction steps (30 min each, CHCl_3_:MeOH; 9:1 v/v). All samples were pre-extracted to remove interfering soil organic matter (30 min, MeOH) before two sequential polymer extraction steps.

Without sample pretreatment, the amounts of PBAT and PLA extracted in both the first and the second extraction step were highly variable (Figure 1, sample pretreatment: “none”). Furthermore, larger amounts of both polyesters were extracted during the second extraction step. These findings show that soil compaction impaired extraction, likely due to slow solvent diffusion through the soil blocks.

Both coarsely crushing and finely grinding the samples before extraction substantially improved the reproducibility of the extraction and increased the amounts of both polymers extracted during the first step (Figure 1, sample pretreatment: “crushing” and “grinding”). For both pretreatments, however, small amounts of PBAT and PLA were present in the second extraction step, implying that a single extraction step of 30 min was insufficient for a complete extraction from the compacted soil.

It is noteworthy that the total amount of PLA extracted over both extraction steps from the finely ground samples matched the amount of PLA initially added to the mesh bags (i.e., 100 ± 5%): all of the PLA was thus extracted, implying not only that PLA did not biodegrade within four months of soil incubation, but also that no PLA losses occurred during the delivery, incubation, retrieval, freeze-drying, and grinding of the samples. This latter finding demonstrates that the mesh bags are an effective mean to deliver and retrieve polymer samples in soil incubations.

In contrast to PLA, the total amount of PBAT extracted from the same samples was only 78 ± 19% of the initial amount (Figure 1). Since PBAT and PLA are uniformly blended into MF-R (see Section 3, SI), this corresponds to an actual decrease in the mass of PBAT in the sample, and, therefore, biodegradation of PBAT.

Based on these results, all samples collected from the mesocosm incubations presented below were manually ground before extraction, pre-extracted to remove SOM (30 min, MeOH) then extracted (CHCl_3_:MeOH) for 60 min in a single step to ensure complete extraction. A model protocol for these extractions is included in Section 7, SI.

#### PBAT, PLA, and PHBH Biodegradation in Soil Mesocosms

The biodegradation of PBAT and PLA from MF-R and of PHBH was assessed in soil mesocosms filled with AGR-1, AGR-2, or AGR-3 for 6 months. Upon retrieval, the samples were processed using the sample preparation method described above, before extraction and quantification by ^1^H-NMR.

**Figure 2:**
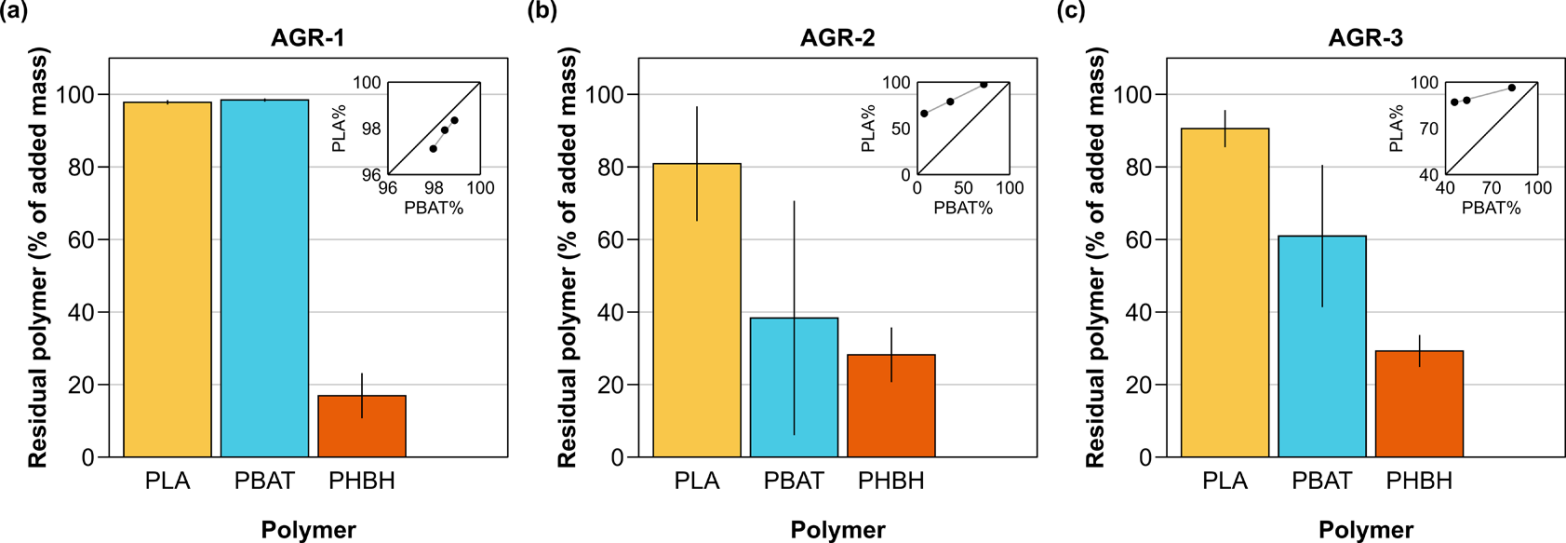
Biodegradation of poly(butylene adipate-*co*-terephthalate) (PBAT) and polylactic acid (PLA), both from biodegradable mulch film MF-R, and of poly(3-hydroxybutyrate-*co*-3-hydroxyhexanoate) (PHBH) after six months of incubation in three different soils: (a) AGR-1, (b) AGR-2, and (c) AGR-3. The individual values of the residual PBAT and PLA masses of each replicate sample are plotted against each other in the insets with the corresponding linear regression (solid line) and the 1:1 line for reference (dashed line). The residual polymer mass is expressed as a percentage of the respective polymer mass initially added to the samples. The heights of the bars represent the average of each group, and the error bars the corresponding standard deviation (*n* = 3). All samples were ground before extraction, pre-extracted for 30 min with MeOH to remove interfering soil organic matter, and then extracted for 60 min with CHCl_3_:MeOH, 9:1 v/v, to extract residual polymer(s).

In all soils tested, the mass of PHBH extracted after six months of incubation was only about 20 to 30% of the mass initially added to the soil (Figure 2). PHBH thus underwent extensive biodegradation, consistent with the high biodegradation rates of polyhydroxyalkanoates reported for different environments, including soils.^23,52−54^ Compared to PHBH, the mass losses of PBAT and PLA, and thus their biodegradation extents, were smaller. In fact, in soil AGR-1, the extracted masses of both PBAT and PLA were in good agreement with their initial masses, implying that neither PBAT nor PLA had biodegraded in this soil. This lack of biodegradation may be linked to the low soil pH and/or a soil microbial community characterized by a low abundance of biodegrading microorganisms.

Compared to soil AGR-1, the masses of PBAT and PLA extracted from AGR-2 and AGR-3 corresponded to only 40 to 60% and to 80 to 90% of their initial masses, respectively. This finding implies that both polymers had biodegraded in these two soils over six months. The more extensive biodegradation of PBAT compared to PLA is consistent with previous studies reporting higher biodegradation rates for the former.^55−59^

Although the difference in the biodegradation extents across soils was significant (weighted least-squares ANOVA, *p* < 0.05) for each polymer, the large variations in the residual masses of PBAT and PLA among triplicate samples imply substantial variations in their biodegradation rates within the same mesocosm, likely reflecting spatial heterogeneity in the incubation conditions (e.g., temperature, soil water content, abundance of microbial degraders). At the same time, the relatively good correlation between the residual masses of PBAT and PLA within each group of replicates (Figure 2, inserts) suggests that this heterogeneity affected the biodegradation rates of both polyesters in a similar manner.

Overall, these results demonstrate that the sample deployment approach presented herein, combined with the analysis of residual polymer with the analytical method developed above, allows monitoring polymer biodegradation in soil incubations outside of the laboratory. As suggested by the large differences in the extent of PBAT and PLA biodegradation observed across the three soils during mesocosm incubations, studies that monitor polymer biodegradation in different soils in the field are needed. During typical farming practices, biodegradable mulch films are applied to agricultural soils repeatedly over the year(s). Predictions of the fate of biodegradable polymers and the potential formation of steady-state concentrations in agricultural soils remain based on laboratory incubation rate data.^60,61^ The workflow presented here can be used to establish robust biodegradation performance of the polymer(s) also in field soils under natural conditions.

## Implications

This work presents an analytical workflow to quantify biodegradable polyesters in soils that can be retraced and validated for additional polymer–soil combinations in future work. While only quantification using ^1^H-NMR was presented herein (achieving a LOD of the order of 10 μg polymer/g soil), the extraction process can be coupled with different analytical techniques (e.g., gas chromatography–mass spectrometry^62^ or pyrolysis-gas chromatography–mass spectrometry), potentially further increasing the method’s sensitivity (but possibly compromising on selectivity). Additionally, the workflow can be coupled with the sample deployment system presented herein to directly monitor polyester biodegradation in the field over time (as showcased herein in a proof-of-concept mesocosm incubations for three agricultural soils). Such field incubation studies are needed to assess and demonstrate the transferability of biodegradation results from highly controlled laboratory incubations (which form the basis for polymer soil biodegradability certification standards) to the actual receiving environment to which the biodegradable polymers are applied (e.g., agricultural fields). We anticipate that the approach developed herein will also be applicable to biodegradation studies in other natural and engineered systems, such as sediments, sludge, and compost,^63^ after appropriate adaptations of the analytical method for the different incubation matrices: all polymers that are chloroform-extractable and show distinct ^1^H-NMR peaks can, in principle, be quantified.

## Supporting Information Available

Detailed soil sampling procedure, field locations, and GPS coordinates (Section 1); chemical structures and reference polymer compositions (Section 2); ^1^H-NMR acquisition parameters, reference spectra, and quantifying equations (Section 3); comprehensive list of R packages used for data analysis (Section 4); ^1^H-NMR spectra of soils and calibration curves for the determination of the LOD (Section 5); polymer recoveries for different extraction times (Section 6); and example of sample preparation and of a complete extraction protocol (Section 7) (PDF)

## Abbreviations

^1^H-NMR: proton nuclear magnetic resonance spectroscopy
BA: 1,4-butanediol–adipic acid repeat unit in PBAT
BT: 1,4-butanediol–terephthalic acid repeat unit in PBAT
CDCl_3_: deuterated chloroform
CHCl_3_: chloroform
DMB: 1,4-dimethoxybenzene
IS: internal standard
LOD: limit of detection
LOQ: limit of quantification
MeOH: methanol
MF-E: biodegradable mulch film ecovio M2351, BASF SE, Germany
MF-R: biodegradable mulch film Bio Mulchfolie 32.00009, gvz-rossat SA, Switzerland
MF-S: biodegradable mulch film Biofolie 15 μ, Sansonnens FG Frères SA, Switzerland
PBA: polybutylene adipate
PBAT: poly(butylene adipate-*co*-terephthalate)
PBAz: polybutylene azelate
PBS: polybutylene succinate
PHBH: poly(3-hydroxybutyrate-*co*-3-hydroxyhexanoate)
PHBV: poly(3-hydroxybutyrate-*co*-3-hydroxyvalerate)
PLA: polylactic acid
PS: polystyrene
SI: Supporting Information
SOM: soil organic matter
TFB: 1,4-bis(trifluoromethyl)benzene

## References

[ref1] European Commission and Directorate-General for Research and Innovation. Biodegradability of Plastics in the Open Environment. Publications Office of the European Union, 2020. 10.2777/690248

[ref2] Paul-Pont I.; Ghiglione J.-F.; Gastaldi E.; Ter Halle A.; Huvet A.; Bruzaud S.; Lagarde F.; Galgani F.; Duflos G.; George M.; Fabre P. Discussion about Suitable Applications for Biodegradable Plastics Regarding Their Sources, Uses and End of Life. Waste Management 2023, 157, 242–248. 10.1016/j.wasman.2022.12.022. 36577275

[ref3] Bauchmüller V.; Carus M.; Chinthapalli R.; Dammer L.; Hark N.; Partanen A.; Ruiz P.; Lajewski S. BioSinn-Products for Which Biodegradation Makes Sense; nova-Institut für politische und ökologische Innovation GmbH: Hürth, Germany, 2021. http://www.nova-institute.eu/biosinn/ (accessed 2025-02-07).

[ref4] Yu Y.; Flury M. Unlocking the Potentials of Biodegradable Plastics with Proper Management and Evaluation at Environmentally Relevant Concentrations. npj Mater. Sustain. 2024, 2 (1), 9 10.1038/s44296-024-00012-0.

[ref5] Sander M. Biodegradation of Polymeric Mulch Films in Agricultural Soils: Concepts, Knowledge Gaps, and Future Research Directions. Environ. Sci. Technol. 2019, 53 (5), 2304–2315. 10.1021/acs.est.8b05208. 30698422

[ref6] Guerrini S.; Borreani G.; Voojis H. Biodegradable Materials in Agriculture: Case Histories and Perspectives. In Soil Degradable Bioplastics for a Sustainable Modern Agriculture; Malinconico M., Ed.; Green Chemistry and Sustainable Technology; Springer: Berlin, 2017; pp 35–65. 10.1007/978-3-662-54130-2_3

[ref7] Mo A.; Zhang Y.; Gao W.; Jiang J.; He D. Environmental Fate and Impacts of Biodegradable Plastics in Agricultural Soil Ecosystems. Applied Soil Ecology 2023, 181, 104667 10.1016/j.apsoil.2022.104667.

[ref8] FAO. Assessment of Agricultural Plastics and Their Sustainability: A Call for Action; 2021; Vol. 9. 10.4060/cb7856en

[ref9] Hemphill D. D. Agricultural Plastics as Solid Waste: What Are the Options for Disposal?. HortTechnology 1993, 3 (1), 70–73. 10.21273/HORTTECH.3.1.70.

[ref10] Sintim H. Y.; Flury M. Is Biodegradable Plastic Mulch the Solution to Agriculture’s Plastic Problem?. Environ. Sci. Technol. 2017, 51 (3), 1068–1069. 10.1021/acs.est.6b06042. 28078891

[ref11] Hofmann T.; Ghoshal S.; Tufenkji N.; Adamowski J. F.; Bayen S.; Chen Q.; Demokritou P.; Flury M.; Hüffer T.; Ivleva N. P.; Ji R.; Leask R. L.; Maric M.; Mitrano D. M.; Sander M.; Pahl S.; Rillig M. C.; Walker T. R.; White J. C.; Wilkinson K. J. Plastics Can Be Used More Sustainably in Agriculture. Communications Earth & Environment 2023, 4 (1), 1–11. 10.1038/s43247-023-00982-4.37325084

[ref12] Graf Y.; Hein S.; Schnabl A. S. A Review of Challenges and Future Pathways for Decision Making with Treeshelters - A German and European Perspective. Journal of Forest Research 2022, 27 (3), 191–199. 10.1080/13416979.2022.2029281.

[ref13] Arnold J. C.; Alston S. M. Life Cycle Assessment of the Production and Use of Polypropylene Tree Shelters. Journal of Environmental Management 2012, 94 (1), 1–12. 10.1016/j.jenvman.2011.09.005. 22098783

[ref14] The Drive to Stop Plastic Pollution Growing in New Forests. BBC News. January 22, 2020. https://www.bbc.com/news/uk-scotland-highlands-islands-51206456 (accessed 2023-10-29).

[ref15] Satti S. M.; Shah A. A. Polyester-based Biodegradable Plastics: An Approach towards Sustainable Development. Letters in Applied Microbiology 2020, 70 (6), 413–430. 10.1111/lam.13287. 32086820

[ref16] Degli-Innocenti F.; Breton T.; Chinaglia S.; Esposito E.; Pecchiari M.; Pennacchio A.; Pischedda A.; Tosin M. Microorganisms That Produce Enzymes Active on Biodegradable Polyesters Are Ubiquitous. Biodegradation 2023, 34 (6), 489–518. 10.1007/s10532-023-10031-8. 37354274

[ref17] Ghosh K.; Jones B. H. Roadmap to Biodegradable Plastics—Current State and Research Needs. ACS Sustainable Chem. Eng. 2021, 9 (18), 6170–6187. 10.1021/acssuschemeng.1c00801.

[ref18] Harrison J. P.; Boardman C.; O’Callaghan K.; Delort A.-M.; Song J. Biodegradability Standards for Carrier Bags and Plastic Films in Aquatic Environments: A Critical Review. Royal Society Open Science 2018, 5 (5), 171792 10.1098/rsos.171792. 29892374 PMC5990801

[ref19] Briassoulis D.; Degli Innocenti F. Standards for Soil Biodegradable Plastics. In Soil Degradable Bioplastics for a Sustainable Modern Agriculture; Malinconico M., Ed.; Green Chemistry and Sustainable Technology; Springer: Berlin, 2017; pp 139–168. 10.1007/978-3-662-54130-2_6

[ref20] Wilde B. D. 5. International and National Norms on Biodegradability and Certification Procedures. In Handbook of Biodegradable Polymers; Bastioli C., Ed.; De Gruyter, 2020; pp 115–146. 10.1515/9781501511967-005

[ref21] Hayes D. G.; Flury M. Summary and Assessment of EN 17033:2018, a New Standard for Biodegradable Plastic Mulch Films. 2018. https://biodegradablemulch.tennessee.edu/wp-content/uploads/sites/214/2020/12/EU-regs-factsheet.pdf.

[ref22] van der Zee M. Analytical Methods for Monitoring Biodegradation Processes of Environmentally Degradable Polymers. In Handbook of Biodegradable Polymers; John Wiley & Sons, Ltd., 2011; pp 263–281. 10.1002/9783527635818.ch11

[ref23] Kim M. S.; Chang H.; Zheng L.; Yan Q.; Pfleger B. F.; Klier J.; Nelson K.; Majumder E. L.-W.; Huber G. W. A Review of Biodegradable Plastics: Chemistry, Applications, Properties, and Future Research Needs. Chem. Rev. 2023, 123 (16), 9915–9939. 10.1021/acs.chemrev.2c00876. 37470246

[ref24] Afshar S. V.; Boldrin A.; Astrup T. F.; Daugaard A. E.; Hartmann N. B. Degradation of Biodegradable Plastics in Waste Management Systems and the Open Environment: A Critical Review. Journal of Cleaner Production 2024, 434, 140000 10.1016/j.jclepro.2023.140000.

[ref25] Zumstein M. T.; Schintlmeister A.; Nelson T. F.; Baumgartner R.; Woebken D.; Wagner M.; Kohler H.-P. E.; McNeill K.; Sander M. Biodegradation of Synthetic Polymers in Soils: Tracking Carbon into CO_2_ and Microbial Biomass. Science Advances 2018, 4 (7), eaas9024 10.1126/sciadv.aas9024. 30050987 PMC6059733

[ref26] Kaplan D. L.; Hartenstein R.; Sutter J. Biodegradation of Polystyrene, Poly(Methyl Methacrylate), and Phenol Formaldehyde. Appl. Environ. Microbiol. 1979, 38 (3), 551–553. 10.1128/aem.38.3.551-553.1979. 533278 PMC243531

[ref27] Eubeler J. P.; Zok S.; Bernhard M.; Knepper T. P. Environmental Biodegradation of Synthetic Polymers I. Test Methodologies and Procedures. TrAC Trends in Analytical Chemistry 2009, 28 (9), 1057–1072. 10.1016/j.trac.2009.06.007.

[ref28] Lucas N.; Bienaime C.; Belloy C.; Queneudec M.; Silvestre F.; Nava-Saucedo J.-E. Polymer Biodegradation: Mechanisms and Estimation Techniques - A Review. Chemosphere 2008, 73 (4), 429–442. 10.1016/j.chemosphere.2008.06.064. 18723204

[ref29] Andrady A. L. Assessment of Environmental Biodegradation of Synthetic Polymers. Journal of Macromolecular Science, Part C 1994, 34 (1), 25–76. 10.1080/15321799408009632.

[ref30] Bläsing M.; Amelung W. Plastics in Soil: Analytical Methods and Possible Sources. Sci. Total Environ. 2018, 612, 422–435. 10.1016/j.scitotenv.2017.08.086. 28863373

[ref31] Papini G.; Petrella G.; Cicero D. O.; Boglione C.; Rakaj A. Identification and Quantification of Polystyrene Microplastics in Marine Sediments Facing a River Mouth through NMR Spectroscopy. Mar. Pollut. Bull. 2024, 198, 115784 10.1016/j.marpolbul.2023.115784. 38016207

[ref32] Günther M.; Imhof W. Highly Selective Solid-Liquid Extraction of Microplastic Mixtures as a Pre-Preparation Tool for Quantitative Nuclear Magnetic Resonance Spectroscopy Studies. Analyst 2024, 149 (24), 5800–5811. 10.1039/D4AN00991F. 39373111

[ref33] Peez N.; Becker J.; Ehlers S. M.; Fritz M.; Fischer C. B.; Koop J. H. E.; Winkelmann C.; Imhof W. Quantitative Analysis of PET Microplastics in Environmental Model Samples Using Quantitative 1H-NMR Spectroscopy: Validation of an Optimized and Consistent Sample Clean-up Method. Anal. Bioanal. Chem. 2019, 411 (28), 7409–7418. 10.1007/s00216-019-02089-2. 31489440

[ref34] Giannattasio A.; Iuliano V.; Oliva G.; Giaquinto D.; Capacchione C.; Cuomo M. T.; Hasan S. W.; Choo K.-H.; Korshin G. V.; Barceló D.; Belgiorno V.; Grassi A.; Naddeo V.; Buonerba A. Micro(Nano)Plastics from Synthetic Oligomers Persisting in Mediterranean Seawater: Comprehensive NMR Analysis, Concerns and Origins. Environ. Int. 2024, 190, 108839 10.1016/j.envint.2024.108839. 38943925

[ref35] Dukek P.; Schleheck D.; Kovermann M. High-Resolution NMR Spectroscopic Approaches to Quantify PET Microplastics Pollution in Environmental Freshwater Samples. Chemosphere 2024, 367, 143657 10.1016/j.chemosphere.2024.143657. 39486629

[ref36] Günther M.; Kirimlioglu Sayilik G.; Imhof W. Determination of Tire Wear Particle-Type Polymers by Combination of Quantitative Nuclear Magnetic Resonance Spectroscopy and Soxhlet Extraction. Molecules 2024, 29 (24), 5899 10.3390/molecules29245899. 39769988 PMC11679811

[ref37] Nelson T. F.; Remke S. C.; Kohler H. P. E.; McNeill K.; Sander M. Quantification of Synthetic Polyesters from Biodegradable Mulch Films in Soils. Environ. Sci. Technol. 2020, 54 (1), 266–275. 10.1021/acs.est.9b05863. 31738056

[ref38] Stadt Zürich. Ökologischer Ausgleich - Steckbriefe zu den anrechenbaren Lebensräumen, 2025. https://www.stadt-zuerich.ch/content/dam/web/de/planen-bauen/bauberatung-und-dienstleistungen/dokumente/gruenraeume-freiraeume/freiraumberatung/oekologischer-ausgleich-erlaeuterungen-steckbriefe-aktualisiert.pdf (accessed 2025-03-13).

[ref39] Soil Science Division Staff. Soil Survey Manual; USDA Handbook 18; U.S. Government Printing Office: Washington, DC, 2017. https://www.nrcs.usda.gov/resources/guides-and-instructions/soil-survey-manual (accessed 2025-02-07).

[ref40] Bharti S. K.; Roy R. Quantitative 1H-NMR Spectroscopy. TrAC Trends in Analytical Chemistry 2012, 35, 5–26. 10.1016/j.trac.2012.02.007.

[ref41] Fulmer G. R.; Miller A. J. M.; Sherden N. H.; Gottlieb H. E.; Nudelman A.; Stoltz B. M.; Bercaw J. E.; Goldberg K. I. NMR Chemical Shifts of Trace Impurities: Common Laboratory Solvents, Organics, and Gases in Deuterated Solvents Relevant to the Organometallic Chemist. Organometallics 2010, 29 (9), 2176–2179. 10.1021/om100106e.

[ref42] R Core Team. R: A Language and Environment for Statistical Computing; R Foundation for Statistical Computing: Vienna, 2024. https://www.R-project.org/ (accessed 2025-02-07).

[ref43] Posit team. RStudio: Integrated Development Environment for R; Posit Software, PBC: Boston, 2024. http://www.posit.co/ (accessed 2025-02-07).

[ref44] Robouch P.; Stroka J.; Haedrich J.; Schaechtele A.; Wenzl T. Guidance Document on the Estimation of LOD and LOQ for Measurements in the Field of Contaminants in Feed and Food. European Commission and Joint Research Centre, Publications Office, 2016. 10.2787/8931

[ref45] Grieder C.; Tanner P. Fact Sheet ARVICOLA Perennial Ryegrass (4n) *Lolium Perenne* L. 2018, 1. https://ira.agroscope.ch/en-US/publication/38965 (accessed 2025-02-07).

[ref46] Larrañaga A.; Lizundia E. A Review on the Thermomechanical Properties and Biodegradation Behaviour of Polyesters. Eur. Polym. J. 2019, 121, 109296 10.1016/j.eurpolymj.2019.109296.

[ref47] Bonartsev A. P.; Boskhomodgiev A. P.; Iordanskii A. L.; Bonartseva G. A.; Rebrov A. V.; Makhina T. K.; Myshkina V. L.; Yakovlev S. A.; Filatova E. A.; Ivanov E. A.; Bagrov D. V.; Zaikov G. E. Hydrolytic Degradation of Pol(3-Hydroxybutyrate), Polylactide and Their Derivatives: Kinetics, Crystallinity, and Surface Morphology. Mol. Cryst. Liq. Cryst. 2012, 556 (1), 288–300. 10.1080/15421406.2012.635982.

[ref48] Udesky J. O.; Dodson R. E.; Perovich L. J.; Rudel R. A. Wrangling Environmental Exposure Data: Guidance for Getting the Best Information from Your Laboratory Measurements. Environmental Health 2019, 18, 99 10.1186/s12940-019-0537-8. 31752881 PMC6868687

[ref49] Linsinger T. P. J. Use of Recovery and Bias Information in Analytical Chemistry and Estimation of Its Uncertainty Contribution. TrAC Trends in Analytical Chemistry 2008, 27 (10), 916–923. 10.1016/j.trac.2008.08.013.

[ref50] Lesnik B. Guidance for Methods Development and Methods Validation for the RCRA Program. Office of Solid Waste, U.S. Environmental Protection Agency: Cincinnatti, OH, 1992; pp 1–32.

[ref51] Rowe B. L.; Delzer G. C.; Bender D. A.; Zogorski J. S. Volatile Organic Compound Matrix Spike Recoveries for Ground-and Surface-Water Samples, 1997–2001. 2005.

[ref52] Volova T. G.; Prudnikova S. V.; Vinogradova O. N.; Syrvacheva D. A.; Shishatskaya E. I. Microbial Degradation of Polyhydroxyalkanoates with Different Chemical Compositions and Their Biodegradability. Microbial Ecology 2017, 73 (2), 353–367. 10.1007/s00248-016-0852-3. 27623963

[ref53] Koller M.; Mukherjee A.; Obruca S.; Zinn M. Polyhydroxyalkanoates (PHA): Microbial Synthesis of Natural Polyesters. In Microbial Production of High-Value Products; Rehm B. H. A., Wibowo D., Eds.; Microbiology Monographs; Springer International Publishing: Cham, Switzerland, 2022; pp 185–236. 10.1007/978-3-031-06600-9_8

[ref54] Amasawa E.; Yamanishi T.; Nakatani J.; Hirao M.; Sato S. Climate Change Implications of Bio-Based and Marine-Biodegradable Plastic: Evidence from Poly(3-Hydroxybutyrate-Co-3-Hydroxyhexanoate). Environ. Sci. Technol. 2021, 55 (5), 3380–3388. 10.1021/acs.est.0c06612. 33586971

[ref55] Brodhagen M.; Peyron M.; Miles C.; Inglis D. A. Biodegradable Plastic Agricultural Mulches and Key Features of Microbial Degradation. Appl. Microbiol. Biotechnol. 2015, 99 (3), 1039–1056. 10.1007/s00253-014-6267-5. 25487893

[ref56] Ren Y.; Hu J.; Yang M.; Weng Y. Biodegradation Behavior of Poly(Lactic Acid) (PLA), Poly(Butylene Adipate-Co-Terephthalate) (PBAT), and Their Blends Under Digested Sludge Conditions. Journal of Polymers and the Environment 2019, 27 (12), 2784–2792. 10.1007/s10924-019-01563-3.

[ref57] Miles C.; DeVetter L.; Ghimire S.; Hayes D. G. Suitability of Biodegradable Plastic Mulches for Organic and Sustainable Agricultural Production Systems. HortScience 2017, 52 (1), 10–15. 10.21273/HORTSCI11249-16.

[ref58] Palsikowski P. A.; Kuchnier C. N.; Pinheiro I. F.; Morales A. R. Biodegradation in Soil of PLA/PBAT Blends Compatibilized with Chain Extender. Journal of Polymers and the Environment 2018, 26, 330–341. 10.1007/s10924-017-0951-3.

[ref59] Satti S. M.; Shah A. A.; Marsh T. L.; Auras R. Biodegradation of Poly(Lactic Acid) in Soil Microcosms at Ambient Temperature: Evaluation of Natural Attenuation, Bio-Augmentation and Bio-Stimulation. Journal of Polymers and the Environment 2018, 26 (9), 3848–3857. 10.1007/s10924-018-1264-x.

[ref60] Pecchiari M.; Degli-Innocenti F.; Tosin M. Biodegradation Rate and Build-up of Plastics in Soil: A Theoretical Approach. Polym. Degrad. Stab. 2024, 228, 110900 10.1016/j.polymdegradstab.2024.110900.

[ref61] Brouwer M. T.; Post W.; van der Zee M.; Reilink R.; Boom R.; Maaskant E. A Predictive Model to Assess the Accumulation of Microplastics in the Natural Environment. Science of The Total Environment 2024, 957, 177503 10.1016/j.scitotenv.2024.177503. 39532184

[ref62] Hernandez-Charpak Y. D.; Kansara H. J.; Lodge J. S.; Eddingsaas N. C.; Lewis C. L.; Trabold T. A.; Diaz C. A. Quantitative Methodology for Poly (Butylene Adipate-Co- Terephthalate) (PBAT) Microplastic Detection in Soil and Compost. Environ. Sci. Pollut. Res. 2025, 10.1007/s11356-025-35978-4. PMC12960377 39888522

[ref63] Steiner T.; Leitner L.-C.; Zhang Y.; Möller J. N.; Löder M. G. J.; Greiner A.; Laforsch C.; Freitag R. Detection and Specific Chemical Identification of Submillimeter Plastic Fragments in Complex Matrices Such as Compost. Sci. Rep. 2024, 14 (1), 2282 10.1038/s41598-024-51185-6. 38280916 PMC10821947

