# Supplementary material for: An Analytical Workflow to Quantify Biodegradable Polyesters in Soils and Its Application to Incubation Experiments
Source: Environ Sci Technol. 2025 Apr 18;59(16):8108–18. doi: 10.1021/acs.est.4c10664 (PMC12044702; doi:10.1021/acs.est.4c10664)
Supplement: Supplementary file 1 — es4c10664_si_001.pdf [file es4c10664_si_001.pdf]

An analytical workflow to quantify biodegradable polyesters in soils and its application to incubation experiments

Supplementary Information

Mattia Cerri[a,δ,❧], *Flora Wille*[a], *Silvan Arn*[a], *Thomas D. Bucheli*[b], *Franco Widmer*[c], *Rhayn Werz*[a,♦], *Kristopher McNeill*[a,‡,*], *Alessandro Manfrin*[a,*], *and Michael Sander*[a,†,*]

[a] Institute of Biogeochemistry and Pollutant Dynamics, Department of Environmental Systems Science, Swiss Federal Institute of Technology Zurich (ETH Zurich), 8006, Zurich, Switzerland

[b] Environmental Analytics, Agroscope, 8046, Zurich, Switzerland

[c] Molecular Ecology, Agroscope, 8046, Zurich, Switzerland.

*Corresponding authors

Number of pages: 55

Number of figures: 24

Number of tables: 16

TABLE OF CONTENTS

SECTION 1. COLLECTION AND CHARACTERIZATION OF SOILS 5
Field locations 5
Soil collection 6
Soil characterization. 7
SECTION 2. POLYMERS AND MULCH FILMS 9
Chemical structures 9
Effective polymer content of pure polymers 11
Polymer composition of MF-R, MF-S, and MF-E 12
Mulch film homogeneity 14
SECTION 3. POLYMER ^{1}H-NMR SPECTRA AND PARAMETERS FOR QUANTIFICATION 16
Acquisition parameters 16
^{1}H-NMR spectra of internal standards 17
1,4-dimethoxybenzene (DMB) 17
1,4-bis(trifluormethyl)benzene (TFB) 18
^{1}H-NMR spectra of pure polymers 19
Polybutylene adipate (PBA) 19
Polybutylene azelate (PBAz) 21
Polybutylene succinate (PBS) 23
Polycaprolactone (PCL) 25
Poly(3-hydroxybutyrate-co-3-hydroxyhexanoate) (PHBH) 27
Poly(3-hydroxybutyrate-co-hydroxyvalerate) (PHBV) 31
Polystyrene (PS) 33
^{1}H-NMR spectra of commercial mulch films 35
MF-R 36
MF-S 37
MF-E 38
SECTION 4. R PACKAGES 41
SECTION 5. EFFECT OF SOM ON POLYMER QUANTIFICATION 42
^{1}H-NMR spectra of soil extracts 42
Effect of the methanol pre-extraction on co-extraction of SOM 44
Calibration curves for the determination of the LOD and LOQ 45
Effect of the number of measurement scans on the LOD 47
Comparison of the LOQ to environmental concentrations 48

SECTION 6. POLYMER RECOVERIES FOR DIFFERENT EXTRACTION TIMES 50
SECTION 7. MODEL PROTOCOL 51
Preparations and sample assembly 51
After incubation 52
Freeze-drying 52
Grinding 52
MeOH pre-extraction step (SOM removal) 52
$CHCl_3$:MeOH extraction step (polymer extraction) 53
Sample reconstitution 53
REFERENCES 55

SECTION 1. COLLECTION AND CHARACTERIZATION OF SOILS

Field locations

The locations and GPS coordinates of the field sites where soils ARG-1, AGR-2, and AGR-3 were collected are shown in Figure S1 and Table S1, respectively.

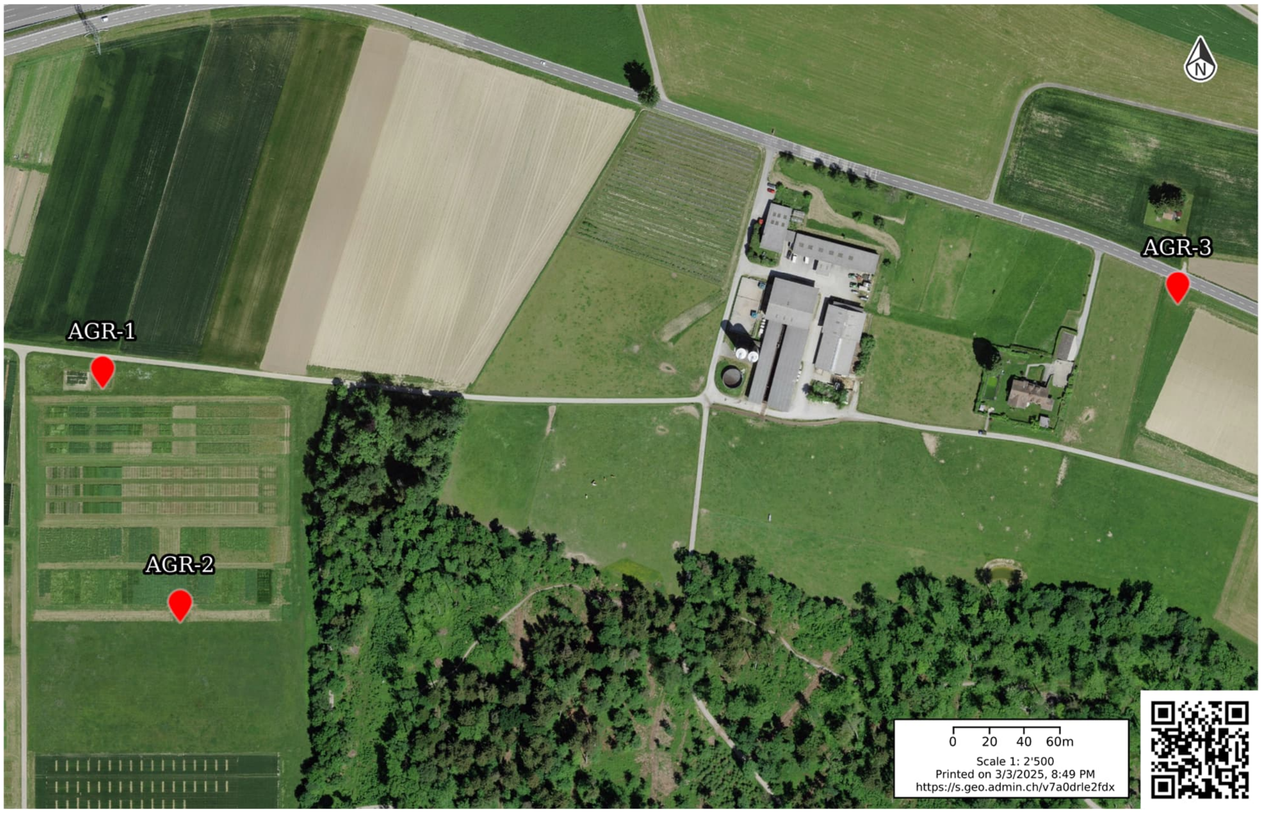

Figure S1. Aerial view of the sites where soils AGR-1, AGR-2, and AGR-3 were collected. The view was exported from the public geographical information portal of the Federal Authorities of the Swiss Confederation, www.geo.admin.ch (©public.geo.admin.ch, CNES, Spot Image, swisstopo).

Table S1. GPS coordinates of the sites where soils AGR-1, AGR-2, and AGR-3 were collected.

**Soil/field site**	**Latitude**	**Longitude**	**Altitude (m AMSL)**
AGR-1	47° 25' 52.5" N	8° 31 24.9" E	445
AGR-2	47° 25' 48.1" N	8° 31 26.9" E	441
AGR-3	47° 25' 53.8" N	8° 31 53.9" E	452

Soil collection

Soils AGR-1, AGR-2, and AGR-3 were collected in May 2020 from the three sites shown in Figure S1. Before collection, the vegetation layers (above-ground grass cover and shallow roots, up to a depth of about 5 cm) were mechanically removed and the soils were thoroughly homogenized up to a depth of approximately 20 cm using a rotary tiller. A large soil sample (~1.5 tons) was collected from each site and then manually homogenized with three cycles of fractional shoveling, each with a 1:5 sampling rate[1]. After this initial homogenization, a subsample of each soil of (~120 kg) was separated from the rest and further homogenized by sieving it down to 2 mm. Any material that did not pass through the sieve was removed. The sieved soil was used to perform all spike-recovery experiments and to fill the mesh bags for the incubations. Part of the remaining homogenized soil (shoveled, but not sieved) was used to fill the mesocosms in which the mesh bags were incubated. Any remaining material (homogenized and sieved) was stored for future studies.

Soil characterization.

The texture of all soils used in this study is shown in Figure S2. Textural classification was based on the United States Department of Agriculture system[2]. Soil characterization, including textural classification and determination of all soil physicochemical parameters listed in Table 1 in the manuscript, was commissioned to LUFA Speyer (Germany). Their standard protocols are referenced in Table S2.

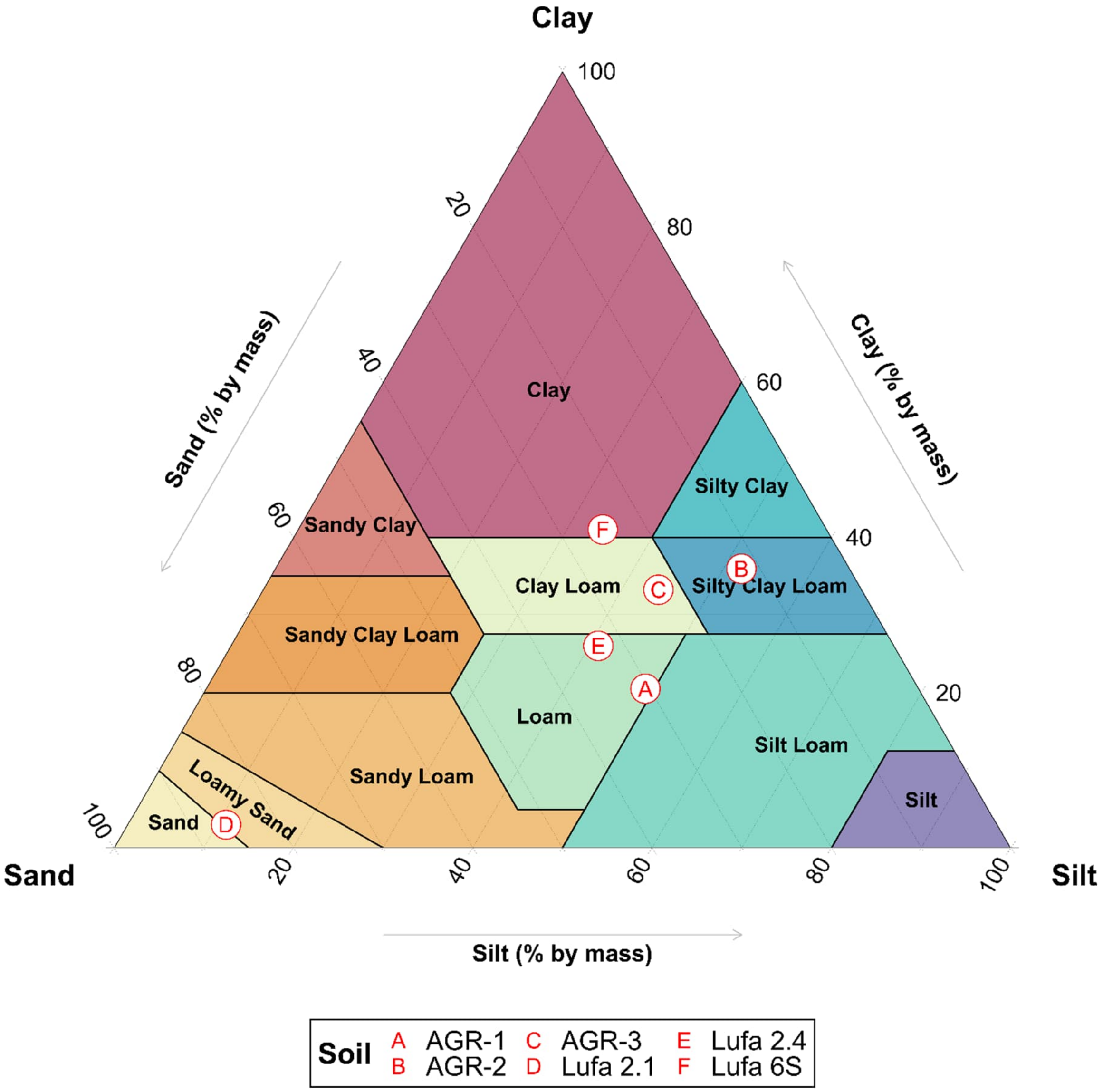

Figure S2. Texture of the six soils used in this study. Textural classification was based on the United States Department of Agriculture system[2].

Table S2. Standard protocols used by LUFA Speyer for soil characterization.

Parameter	Protocol	Reference
Sand content		
Silt content	DIN 11277:2002	[3]
Clay content		
pH	VDLUFA I, A5.1.1:1991	
Total organic carbon	VDLUFA I, A4.1.3.1:2016	[4]
Total nitrogen	VDLUFA I A2.2.5:2011	

SECTION 2. POLYMERS AND MULCH FILMS

Chemical structures

The chemical structures of all polymers included in this study are shown in Table S3.

Table S3. Chemical structures of all polymers used in this study. Images were generated with ACD/ChemSketch (Freeware) 2021.1.3.

Polymer	Chemical structure
Polybutylene adipate (PBA)	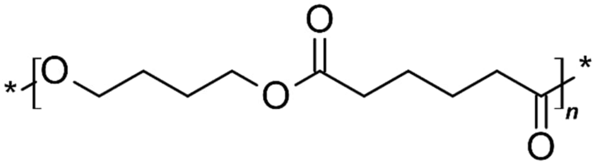
Polybutylene azelate (PBAz)	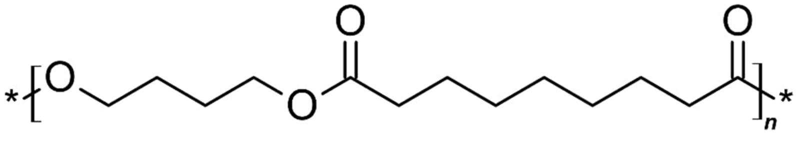
Polybutylene succinate (PBS)	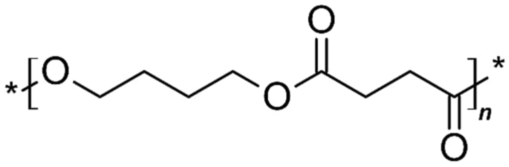
Poly(butylene adipate-co-terephthalate) (PBAT)	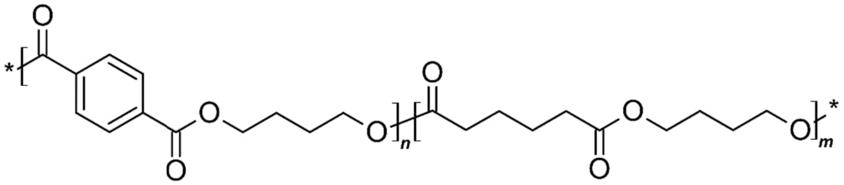

Polycaprolactone<br>(PCL)	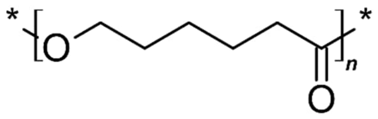
Poly(3-hydroxybutyrate-co-3-hydroxyhexanoate)<br>(PHBH)	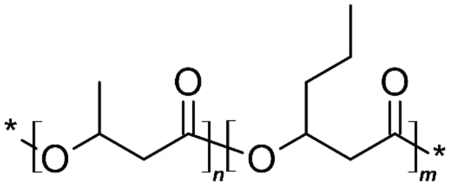
Poly(3-hydroxybutyrate-co-3-hydroxyvalerate)<br>(PHBV)	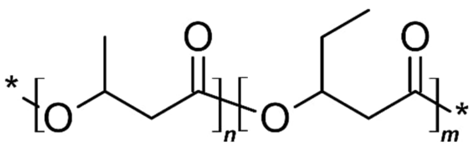
Polylactic acid<br>(PLA)	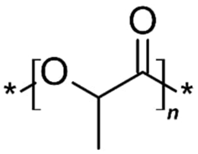
Polystyrene<br>(PS)	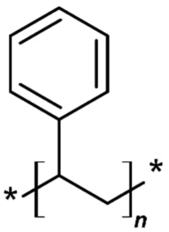

Effective polymer content of pure polymers

The effective polymer content (i.e., purity) of all polymers used in this study is shown in Table S4. The polymer contents were determined by dissolving known amounts of each product (as received by the suppliers) in deuterated chloroform ($CDCl_3$) with known amounts of internal standard and quantifying the corresponding mass of polymer by ^{1}H-NMR.

Table S4. Effective polymer content of all polymers used in this study.

**Polymer**	**Effective polymer content (% by mass)**
PBA	96
PBAz	92
PBS	96
PCL	100
PHBH	97<br>of which { 91% 3-hydroxybutyrate; 9% 3-hydroxyhexanoate }
PHBV	85<br>of which { 93% 3-hydroxybutyrate; 7% 3-hydroxyvalerate }
PS	98

Polymer composition of MF-R, MF-S, and MF-E

The polymer composition of the three mulch films used in this study is shown in Table S5. The mulch films were composed mainly of PBAT and PLA. The remaining components (possibly including plasticizers, organic and inorganic fillers, and UV-stabilizers) were not characterized further. Polymer composition was estimated by dissolving known amounts of each mulch film (as received by the suppliers) in $CDCl_3$ with known amounts of internal standard and quantifying the mass of each polymer by ^{1}H-NMR. Since PBAT is a random copolymer with two diol-diacid repeat units (1,4-butanediol–adipic acid, BA, dimers and 1,4-butanediol–terephthalic acid, BT, dimers), the molar percentage of terephthalic acid over total diacids was also quantified for each mulch film, as defined by Eq. S1:

$$T\% = 100 \cdot \frac{n_T}{n_A + n_T} \qquad \text{Eq. S1}$$

where T% is the molar percentage of terephthalic acid over total diacids in PBAT, and n_T and n_A are the number of moles of terephthalic and adipic acid (or, equivalently, of BA dimers and BT dimers) in the polymer, respectively.

Table S5. Compositions of poly(butylene adipate-co-terephthalate) (PBAT) and polylactic acid (PLA) in the three mulch films MF-R, MF-S, and MF-E and molar percentage of terephthalic acid over total diacids of their PBAT component (indicated as T%, see Eq. S1). All values are reported as mean ± sd, n=45.

**Mulch film**	**PBAT (% by mass)**	**PLA (% by mass)**	**T% of PBAT (molar %)**
MF-R	56 ± 1	14 ± 1	47 ± 1
MF-S	67 ± 1	4 ± 1	47 ± 1
MF-E	67 ± 1	7 ± 1	47 ± 1

Mulch film homogeneity

To confirm the homogeneous distribution of the PBAT and PLA components in MF-R, MF-S and MF-E, a large stretch of each film (1.2 m × 2.5 m) was cut and sub-sampled at regular intervals by punching out discs with a 35 mm diameter stainless steel pritchel. Each disc was dissolved in $CDCl_3$ and its PBAT and PLA content was quantified by ^{1}H-NMR. The semivariograms of the mass percentages of PBAT and PLA were calculated using Matheron's method of moments estimator[5]:

$$\gamma(\mathbf{d}) = \frac{1}{2n(\mathbf{d})}\sum_{i=1}^{n(\mathbf{d})}[z(\mathbf{x_i}) - z(\mathbf{x_i}+\mathbf{d})]^2 \qquad \text{Eq. S2}$$

where $z(\mathbf{x_i})$ and $z(\mathbf{x_i}+\mathbf{d})$ are the observed values of the quantity z (in our case, the mass percentage of PBAT or PLA in the films) at points x_i and $\mathbf{x_i}+\mathbf{d}$, and $n(\mathbf{d})$ is the number of paired comparisons at distance **d**.

The semivariograms obtained for MF-R are shown in Figure S3 (data for MF-S and MF-E not shown). No relevant trends in the spatial distributions of PBAT and PLA were observed within the size of the films that was sampled. The polymer composition of the films was, therefore, considered homogeneous for all experiments, with reference values for the pristine materials shown in Table S5.

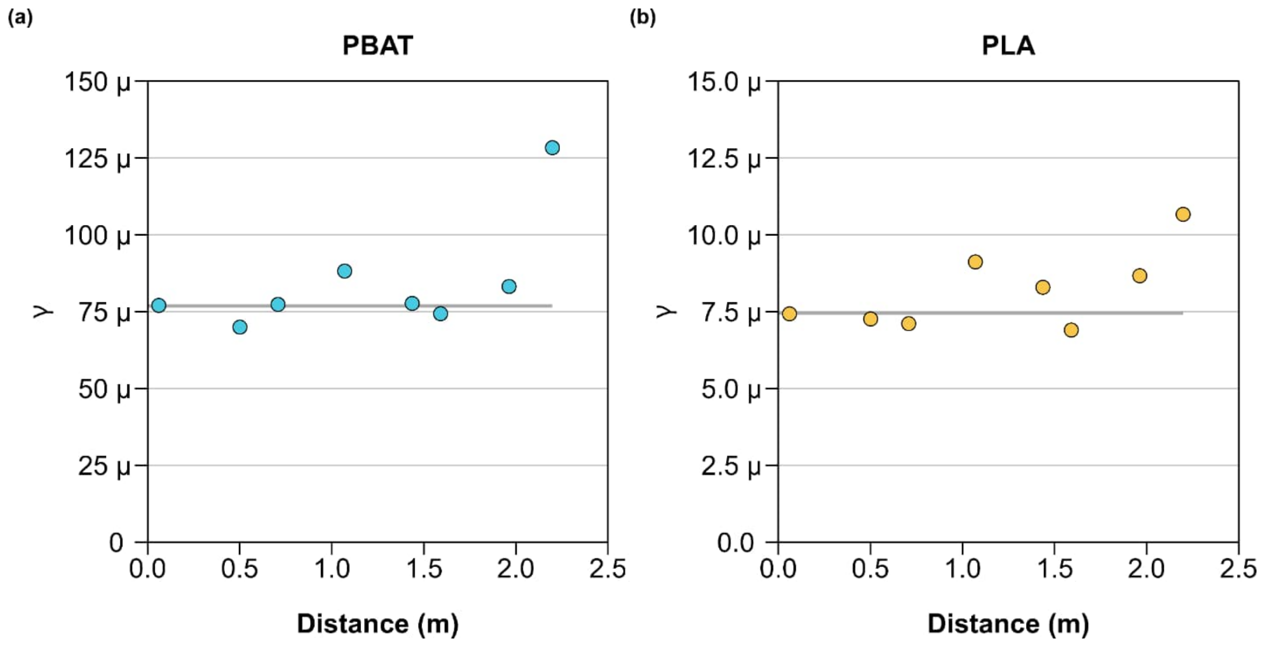

Figure S3. Semivariograms of the mass percentage of (a) poly(butylene adipate-co-terephthalate) (PBAT) and (b) polylactic acid (PLA) across a stretch of MF-R. Points represent the semivariances at increasing distance, while the grey lines are the estimates of the semivariograms nuggets.

SECTION 3. POLYMER ^{1}H-NMR SPECTRA AND PARAMETERS FOR QUANTIFICATION

Acquisition parameters

All measurements were performed with a Bruker Avance III 400 MHz NMR instrument equipped with a Bruker 5 mm BBFO 400 MHz Z gradient probe. Spectra were acquired with the following acquisition parameters: 16 dummy scans and 128 measurement scans with a pulse width of 4.62 μs, a pulse power of 16.795 W, a 15 s delay time between scans and an acquisition time of 4.62 s, with constant spinning of the samples.

The linearity, accuracy and unbiasedness of the NMR response under these conditions have been previously established[6].

^{1}H-NMR spectra of internal standards

The reference spectra of the two internal standards used for quantification are shown in Figure S4 and Figure S5.

1,4-dimethoxybenzene (DMB)

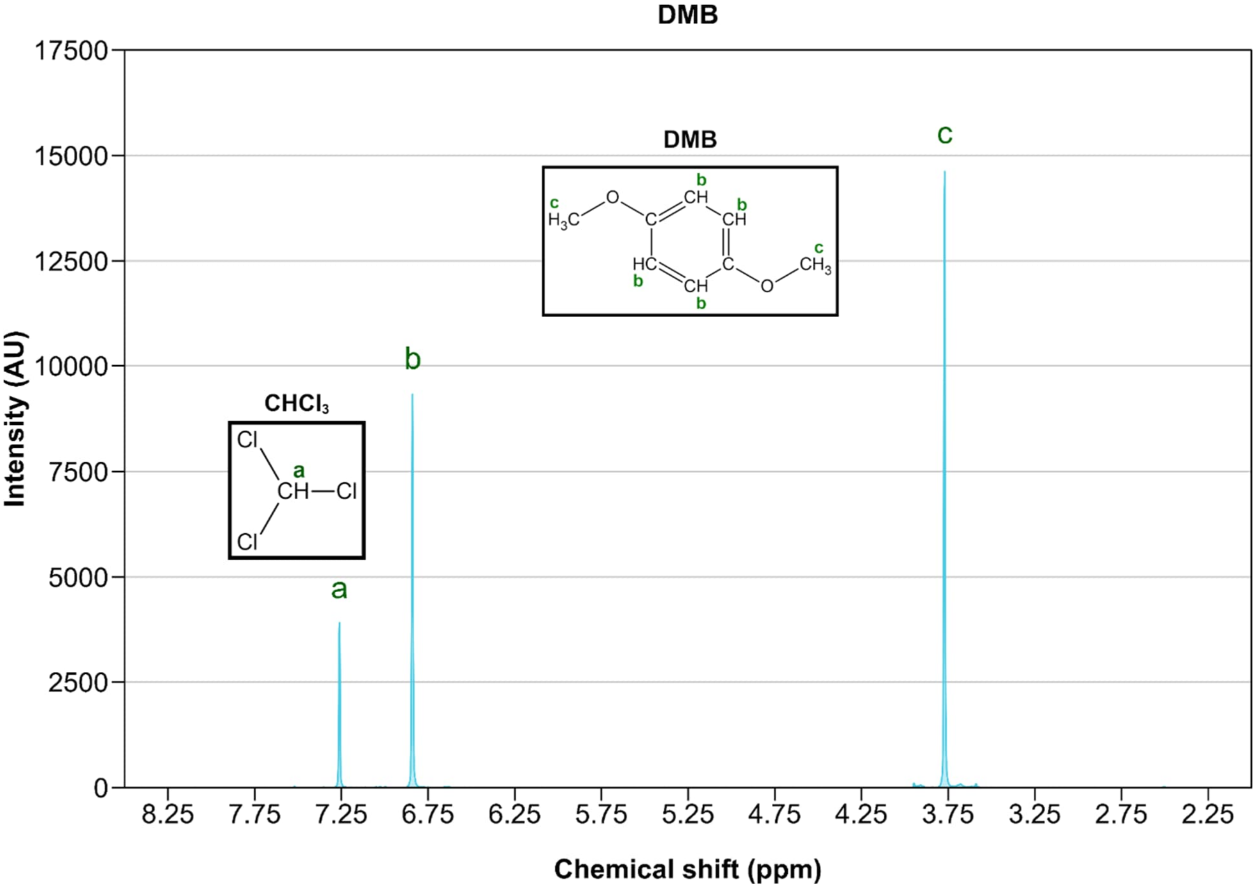

Figure S4. Annotated ^{1}H-NMR spectrum and chemical structure of 1,4-dimethoxybenzene (DMB) used as internal standard for the quantification of all polymers except polystyrene. The spectrum was collected in deuterated chloroform. Peak a is the signal from the traces of non-deuterated chloroform ($CHCl_3$, chemical structure also shown in insert).

1,4-bis(trifluormethyl)benzene (TFB)

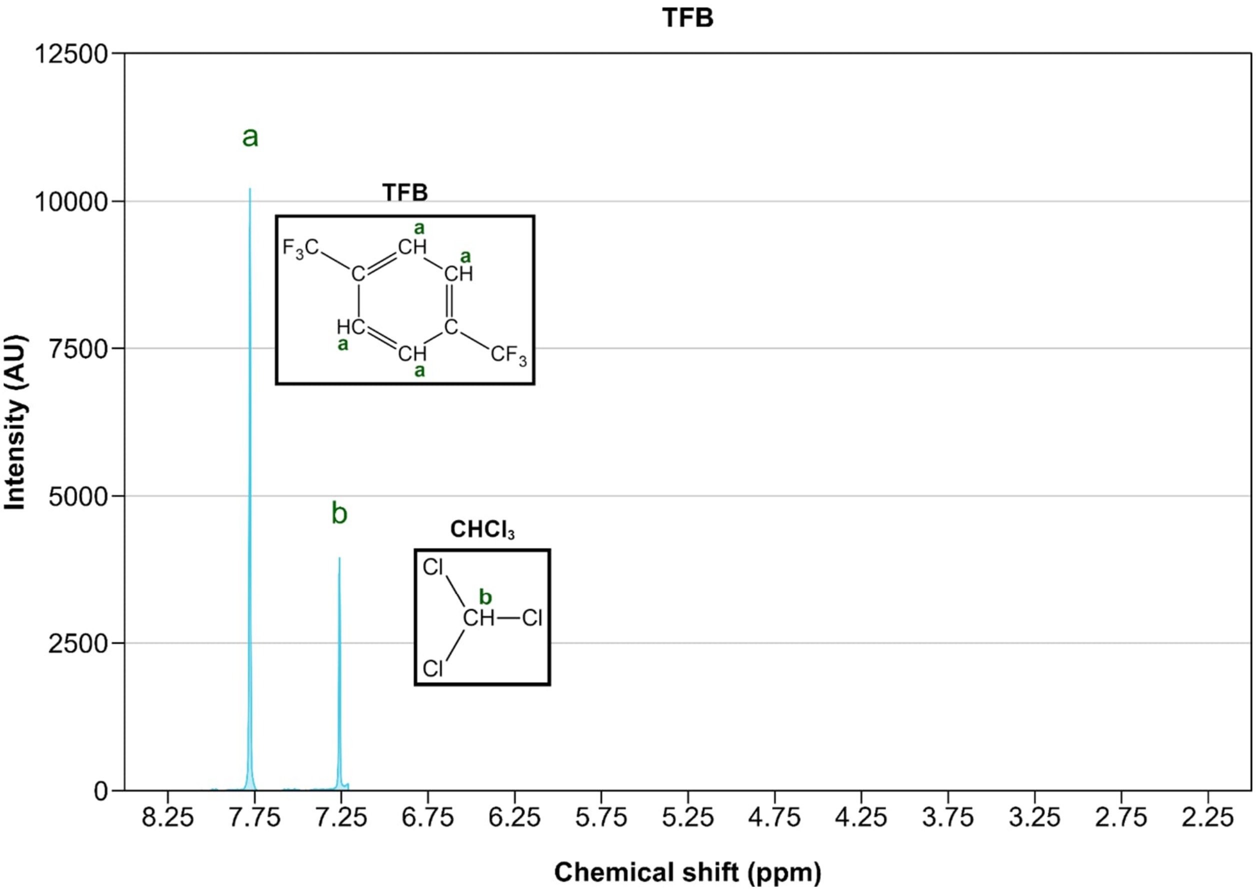

Figure S5. Annotated ^{1}H-NMR spectrum and chemical structure of 1,4-bis(trifluoromethyl)benzene (TFB) used as internal standard for the quantification of polystyrene. The spectrum was collected in deuterated chloroform. Peak b is the signal from the traces of non-deuterated chloroform ($CHCl_3$, chemical structure also shown in insert).

^{1}H-NMR spectra of pure polymers

The reference spectra of all polymers included in this study are shown in Figure S6 to Figure S13. The parameters in Eq.1 (manuscript) for the quantification of each polymer are shown in Table S6 to Table S12.

Polybutylene adipate (PBA)

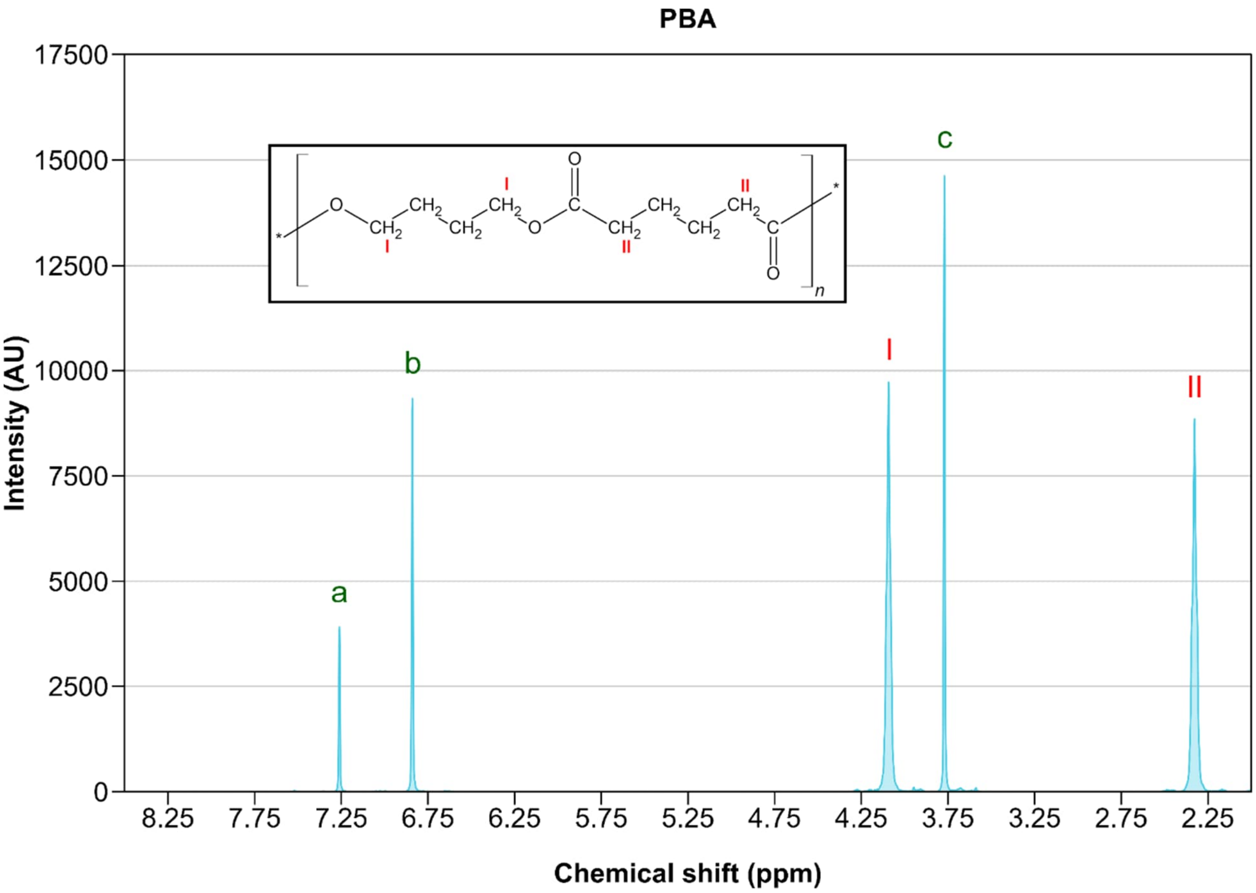

Figure S6. Annotated ^{1}H-NMR spectrum and chemical structure of polybutylene adipate (PBA). The spectrum was collected in deuterated chloroform. Peak a is the signal from the traces of non-deuterated chloroform in the deuterated solvent and peaks b and c are the signals from the internal standard, 1,4-dimethoxybenzene (see Figure S4).

Table S6. Parameters in Eq.1 (manuscript) for the quantification of polybutylene adipate (PBA). Peaks are annotated as shown in Figure S6.

Characteristic peak selected for quantification of the repeat unit	I, chemical shift: ~ 4.08 ppm
$\#H_I$	4 protons/repeat unit
Characteristic peak selected for the IS	**b**, chemical shift: ~ 6.84 ppm
$\#H_{IS}$	4 protons/DMB molecule
Resulting form of Eq.1 (manuscript)	$m_{PBA} = M_{w_{PBA\ unit}} \frac{a_I}{a_{IS}} n_{IS}$
$M_{w_{PBA\ unit}}$	200.06 g/mol

Polybutylene azelate (PBAz)

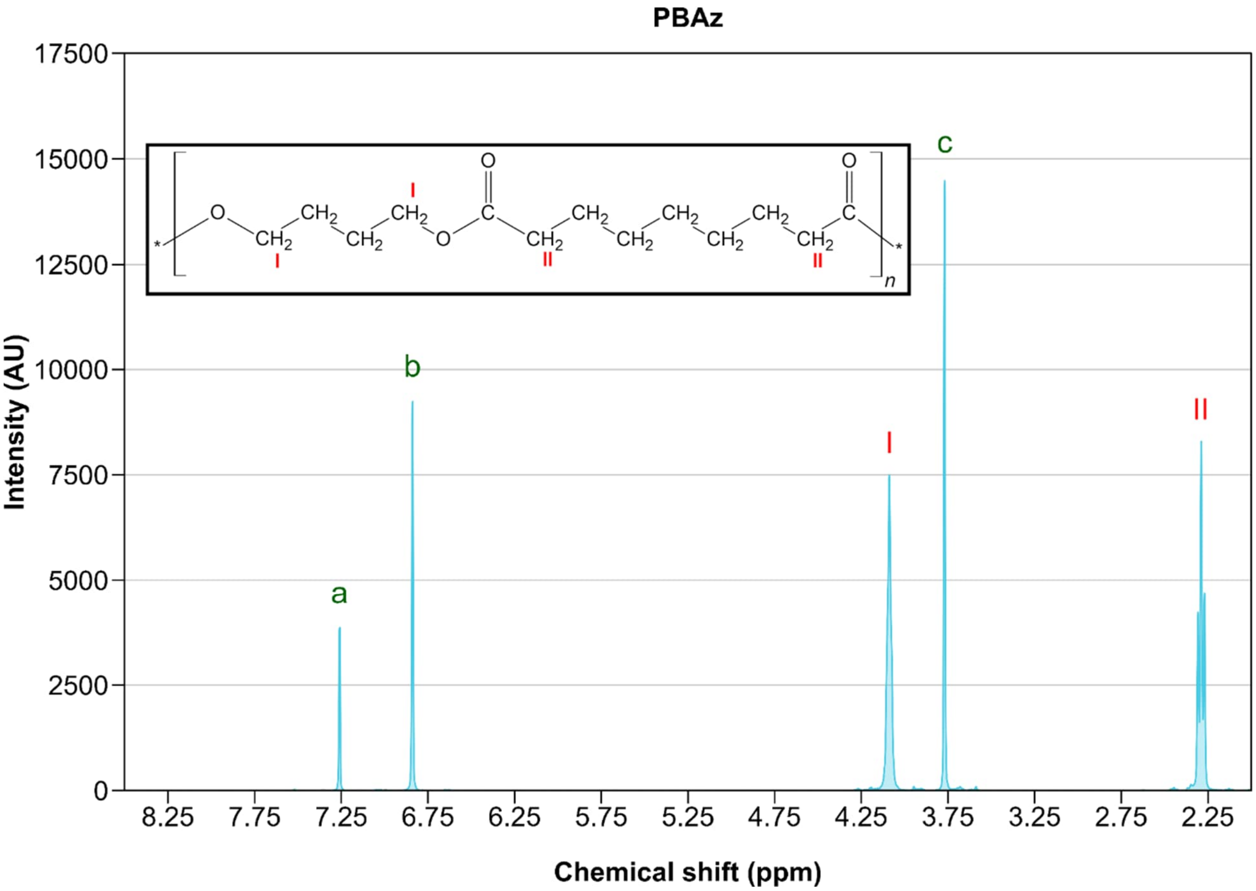

Figure S7. Annotated ^{1}H-NMR spectrum and chemical structure of polybutylene azelate (PBAz). The spectrum was collected in deuterated chloroform. Peak a is the signal from the traces of non-deuterated chloroform in the deuterated solvent and peaks b and c are the signals from the internal standard, 1,4-dimethoxybenzene (see Figure S4).

Table S7. Parameters in Eq.1 (manuscript) for the quantification of polybutylene azelate (PBAz). Peaks are annotated as shown in Figure S7.

Characteristic peak selected for quantification of the repeat unit	I, chemical shift: ~ 4.08 ppm
$\#H_I$	4 protons/repeat unit
Characteristic peak selected for the IS	**b**, chemical shift: ~ 6.84 ppm
$\#H_{IS}$	4 protons/DMB molecule
Resulting form of Eq.1 (manuscript)	$m_{PBAz} = M_{w_{PBAz\ unit}} \frac{a_I}{a_{IS}} n_{IS}$
$M_{w_{PBAz\ unit}}$	242.32 g/mol

Polybutylene succinate (PBS)

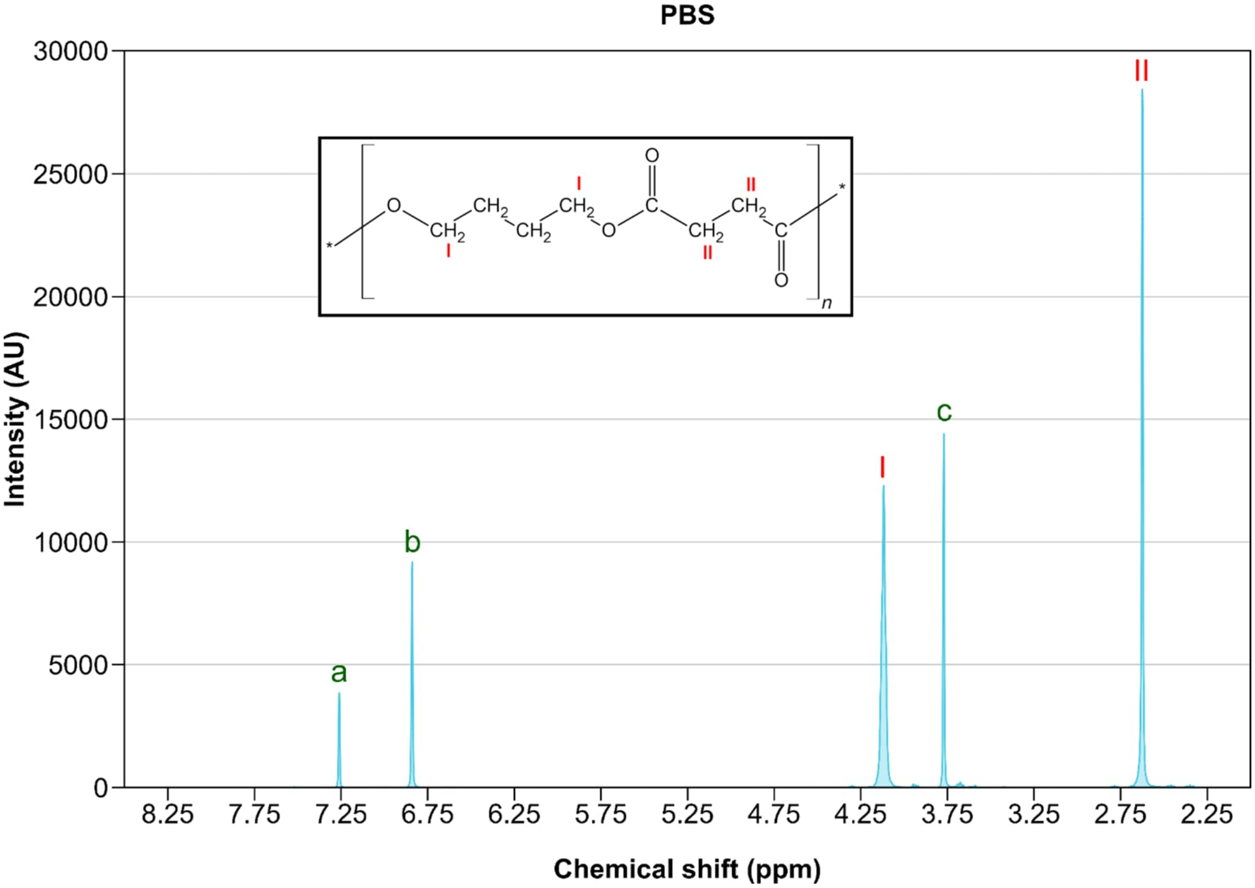

Figure S8. Annotated ^{1}H-NMR spectrum and chemical structure of polybutylene succinate (PBS). The spectrum was collected in deuterated chloroform. Peak a is the signal from the traces of non-deuterated chloroform in the deuterated solvent and peaks b and c are the signals from the internal standard, 1,4-dimethoxybenzene (see Figure S4).

Table S8. Parameters in Eq.1 (manuscript) for the quantification of polybutylene succinate (PBS).

Peaks are annotated as shown in Figure S8.

Characteristic peak selected for quantification of the repeat unit	I, ~ 4.12 ppm
$\#H_I$	4 protons/repeat unit
Characteristic peak selected for the IS	**b**, ~ 6.84 ppm
$\#H_{IS}$	4 protons/DMB molecule
Resulting form of Eq.1 (manuscript)	$m_{PBS} = M_{w_{PBS\ unit}} \frac{a_I}{a_{IS}} n_{IS}$
$M_{w_{PBS\ unit}}$	172.18 g/mol

Polycaprolactone (PCL)

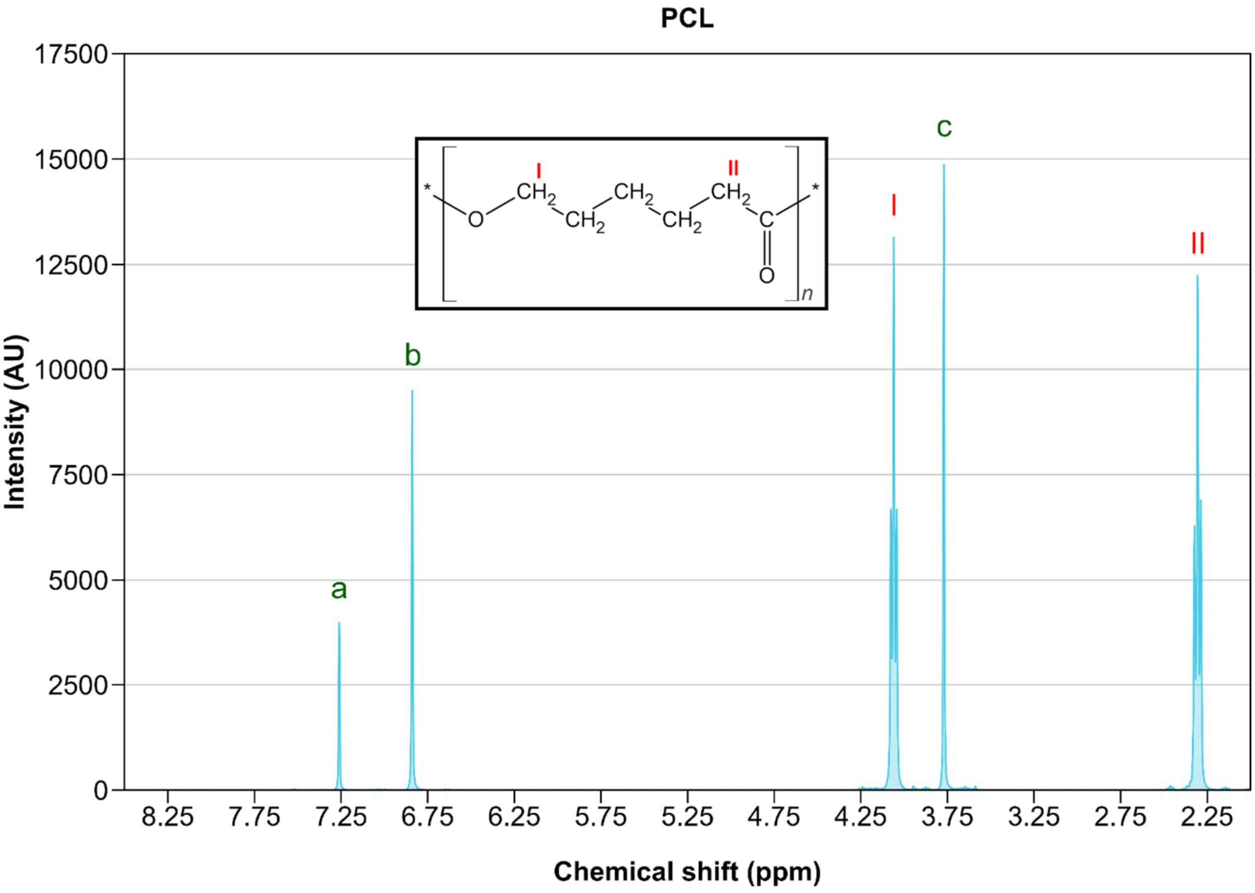

Figure S9. Annotated ^{1}H-NMR spectrum and chemical structure of polycaprolactone (PCL). The spectrum was collected in deuterated chloroform. Peak a is the signal from the traces of non-deuterated chloroform in the deuterated solvent and peaks b and c are the signals from the internal standard, 1,4-dimethoxybenzene (see Figure S4).

Table S9. Parameters in Eq.1 (manuscript) for the quantification of polycaprolactone (PCL). Peaks are annotated as shown in Figure S9.

Characteristic peak selected for quantification of the repeat unit	I, ~ 4.06 ppm
$\#H_I$	2 protons/repeat unit
Characteristic peak selected for the IS	**b**, ~ 6.84 ppm
$\#H_{IS}$	4 protons/DMB molecule
Resulting form of Eq.1 (manuscript)	$m_{PCL} = M_{w_{PCL\ unit}} 2 \frac{a_I}{a_{IS}} n_{IS}$
$M_{w_{PCL\ unit}}$	114.14 g/mol

Poly(3-hydroxybutyrate-co-3-hydroxyhexanoate) (PHBH)

The use of Eq.1 (manuscript) for the quantification of PHBH (and PHBV, see below) requires some additional considerations:

- PHBH is a random copolymer containing two repeat units, 3-hydroxybutyrate (But unit) and 3-hydroxyhexanoate (Hex unit). The masses of both units need to be quantified and added to obtain the total mass of PHBH.
- For some of the soils used in this study, co-extracted soil organic matter (SOM) introduces signals in the ^{1}H-NMR spectrum that overlap with the peaks of PHBH with a chemical shift below 2 ppm (broad, non-specific background signal from SOM) and with peak I in Figure S10 (a quadruplet peak with a chemical shift of ~5.26 ppm whose origin is unknown). Therefore, these peaks could not be used for the quantification of PHBH extracted from soils.
- The area of peak II in Figure S10 can be used to quantify the mass of the But unit, based on Eq.1 (manuscript). This peak is half of the (symmetric) signal resulting from the 2 α protons of the 3-hydroxybutyrate moiety of PHBH (the other half is one of the signals contributing to peak II+III, see next point).
- Peak II+III in Figure S10 is the signal resulting from the superposition of the other symmetric half of peak II and the signal of the 2 α protons of the 3-hydroxyhexanoate moiety of PHBH. Thus, the area of peak II+III can be used to quantify the mass of the Hex unit, after correcting for this superposition, according to Eq. S3. The division of the integration regions of peak II and II+III is shown in Figure S11.

$$m_{Hex} = M_{w_{Hex\ unit}} \frac{\#H_{IS}}{\#H_{Hex,II+III}} \frac{\left(a_{II+III} - \frac{\#H_{But,II+III}}{\#H_{But,II}} a_{II}\right)}{a_{IS}} n_{IS} \qquad \text{Eq. S3}$$

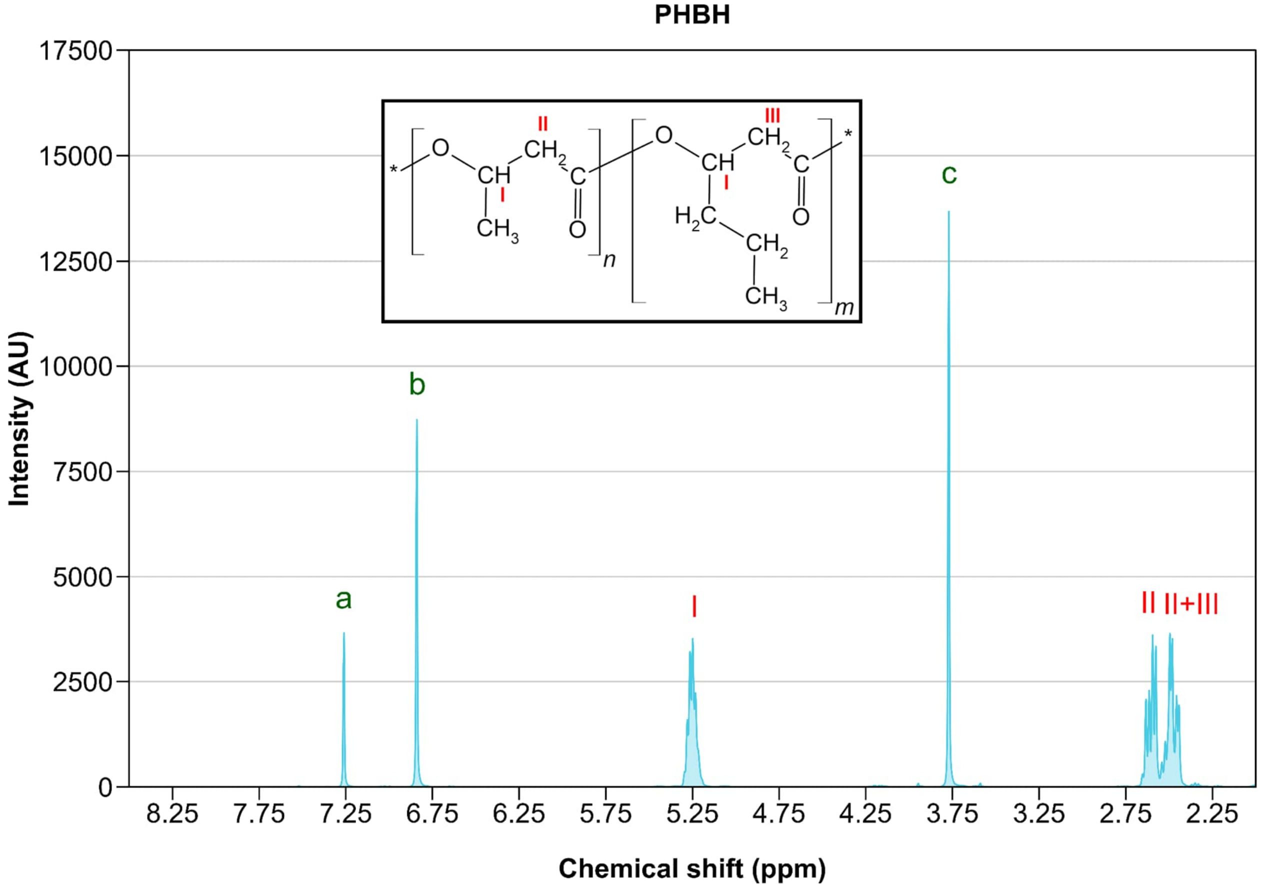

Figure S10. Annotated ^{1}H-NMR spectrum and chemical structure of poly(3-hydroxybutyrate-co-hydroxyhexanoate) (PHBH). The spectrum was collected in deuterated chloroform. Peak a is the signal from the traces of non-deuterated chloroform in the deuterated solvent and peaks b and c are the signals from the internal standard, 1,4-dimethoxybenzene (see Figure S4).

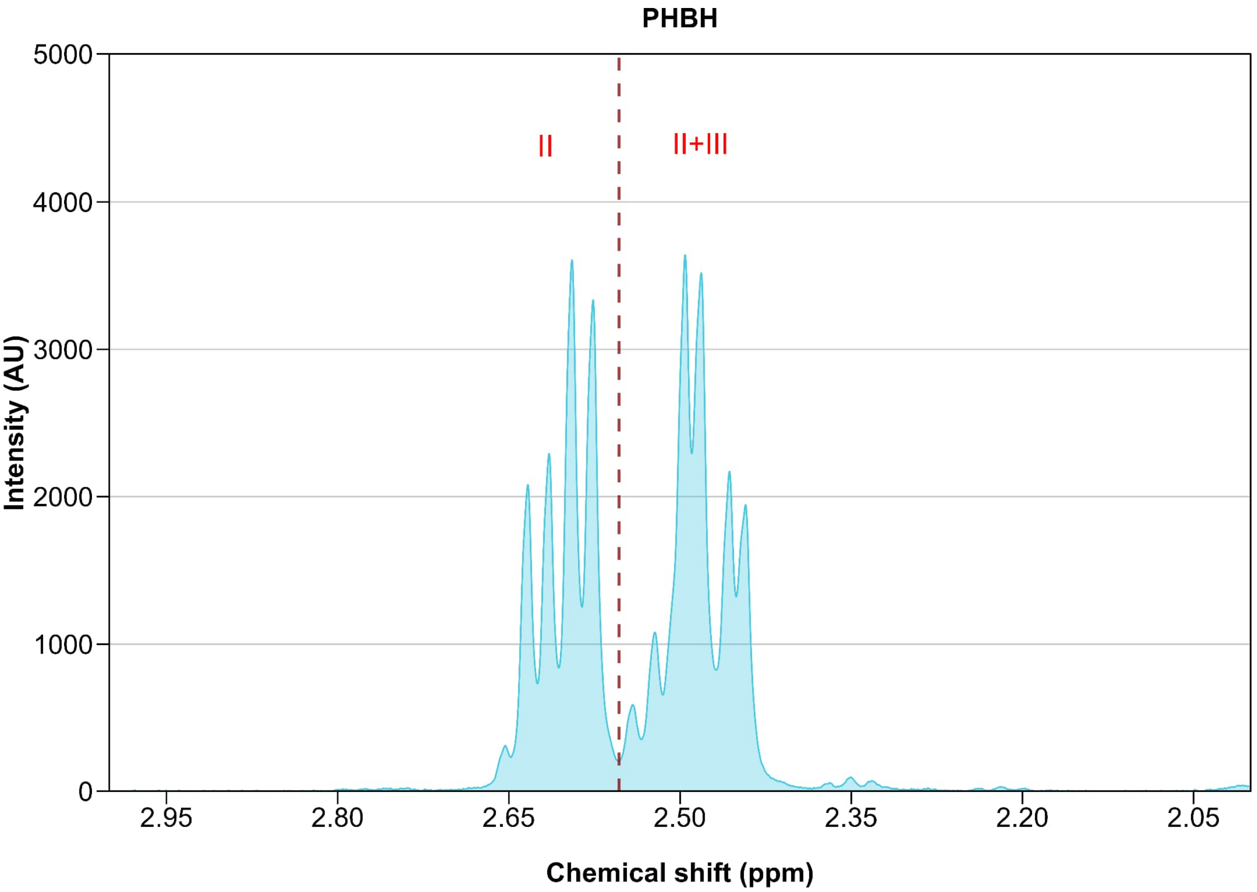

Figure S11. ^{1}H-NMR spectrum of poly(3-hydroxybutyrate-co-hydroxyhexanoate) (PHBH). The spectrum was collected in deuterated chloroform. The dashed vertical line (chemical shift ~2.55 ppm) delimits the integration regions of peaks II and II+III for Eq. S3.

Table S10. Parameters in Eq.1 (manuscript) for the quantification of poly(3-hydroxybutyrate-co-3-hydroxyhexanoate) (PHBH). Peaks are annotated as shown in Figure S10.

Characteristic peak selected for quantification of the But repeat unit	II, chemical shift: ~ 2.62 ppm
$\#H_{But,\ II}$	1 proton/repeat unit (note that the integration limits split the contribution of 2 protons between peak II and peak II+III, see Figure S11)
Characteristic peak selected for quantification of the Hex repeat unit	II+III, chemical shift: ~ 2.48 ppm
$\#H_{But,\ II+III}$	1 proton/repeat unit (note that the integration limits split the contribution of 2 protons between peak II and peak II+III, see Figure S11)
$\#H_{Hex,\ II+III}$	2 protons/repeat unit
Characteristic peak selected for the IS	**b**, chemical shift: ~ 6.84 ppm
$\#H_{IS}$	4 protons/DMB molecule
Resulting form of Eq.1 (manuscript) and Eq. S3	$m_{PHBH} = 2\left[2M_{w_{But\ unit}}a_{II} + M_{w_{Hex\ unit}}(a_{II+III} - a_{II})\right]\frac{n_{IS}}{a_{IS}}$
$M_{w_{But\ unit}}$	86.09 g/mol
$M_{w_{Hex\ unit}}$	114.14 g/mol

Poly(3-hydroxybutyrate-co-hydroxyvalerate) (PHBV)

The same considerations described above for PHBH apply to the quantification of PHBV and the corresponding 3-hydroxybutyrate (But unit) and 3-hydroxyvalerate (Val unit). The division of the integration regions of peak II and II+III in Figure S12 for PHBV is identical to the case of PHBH shown in Figure S11.

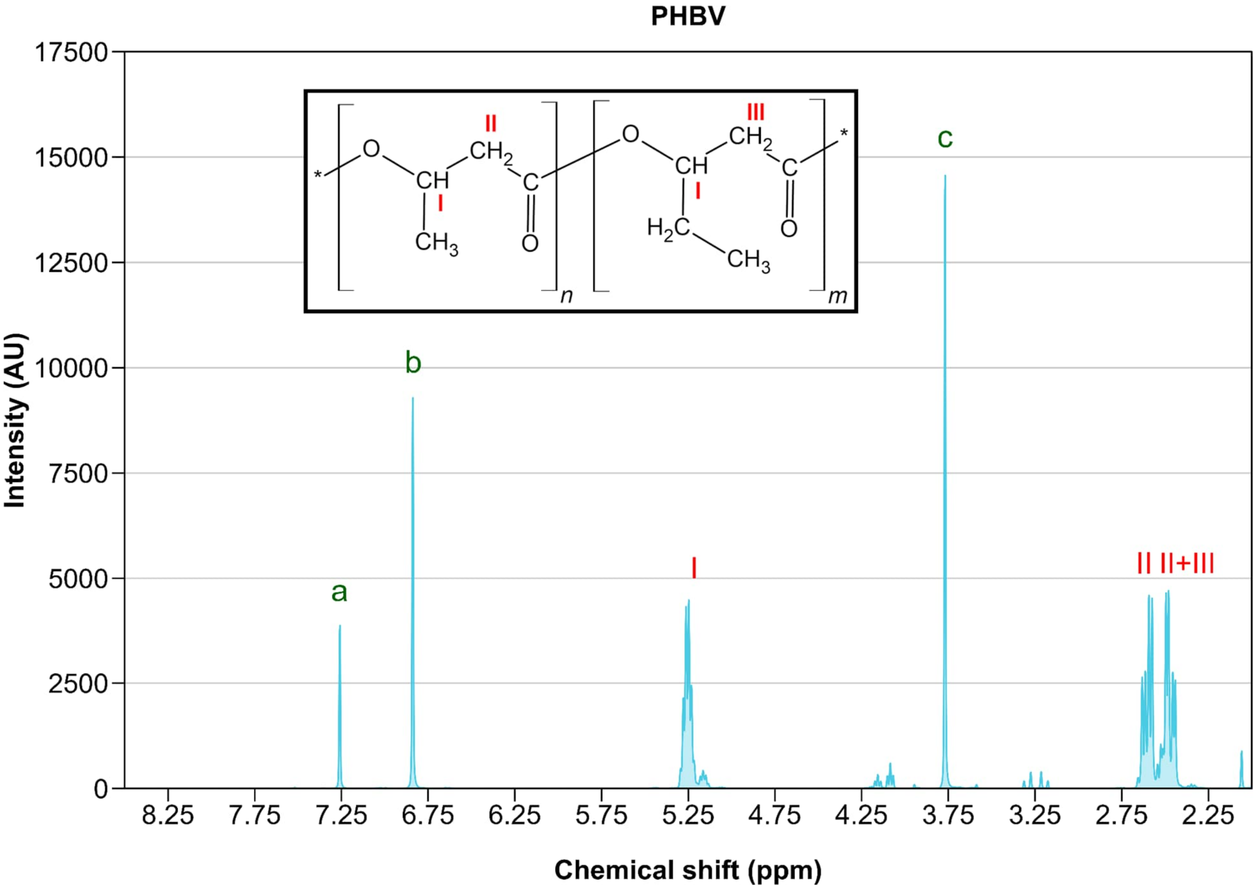

Figure S12. Annotated ^{1}H-NMR spectrum and chemical structure of poly(3-hydroxybutyrate-co-3-hydroxyvalerate) (PHBV). The spectrum was collected in deuterated chloroform. Peak a is the signal from the traces of non-deuterated chloroform in the deuterated solvent and peaks b and c are the signals from the internal standard, 1,4-dimethoxybenzene (see Figure S4).

Table S11. Parameters in Eq.1 (manuscript) for the quantification of poly(3-hydroxybutyrate-co-3-hydroxyvalerate) (PHBV). Peaks are annotated as shown in Figure S12.

Characteristic peak selected for quantification of the But repeat unit	II, chemical shift: ~ 2.62 ppm
$\#H_{But,\ II}$	1 proton/repeat unit (note that the integration limits split the contribution of 2 protons between peak II and peak II+III, see Figure S11)
Characteristic peak selected for quantification of the Val repeat unit	II+III, chemical shift: ~ 2.48 ppm
$\#H_{But,\ II+III}$	1 proton/repeat unit (note that the integration limits split the contribution of 2 protons between peak II and peak II+III, see Figure S11)
$\#H_{Val,\ II+III}$	2 protons/repeat unit
Characteristic peak selected for the IS	**b**, chemical shift: ~ 6.84 ppm
$\#H_{IS}$	4 protons/DMB molecule
Resulting form of Eq.1 (manuscript) and Eq. S3	$m_{PHBV} = 2\left[2M_{w_{But\ unit}} a_{II} + M_{w_{Val\ unit}} (a_{II+III} - a_{II})\right] \frac{n_{IS}}{a_{IS}}$
$M_{w_{But\ unit}}$	86.09 g/mol
$M_{w_{Val\ unit}}$	100.12 g/mol

Polystyrene (PS)

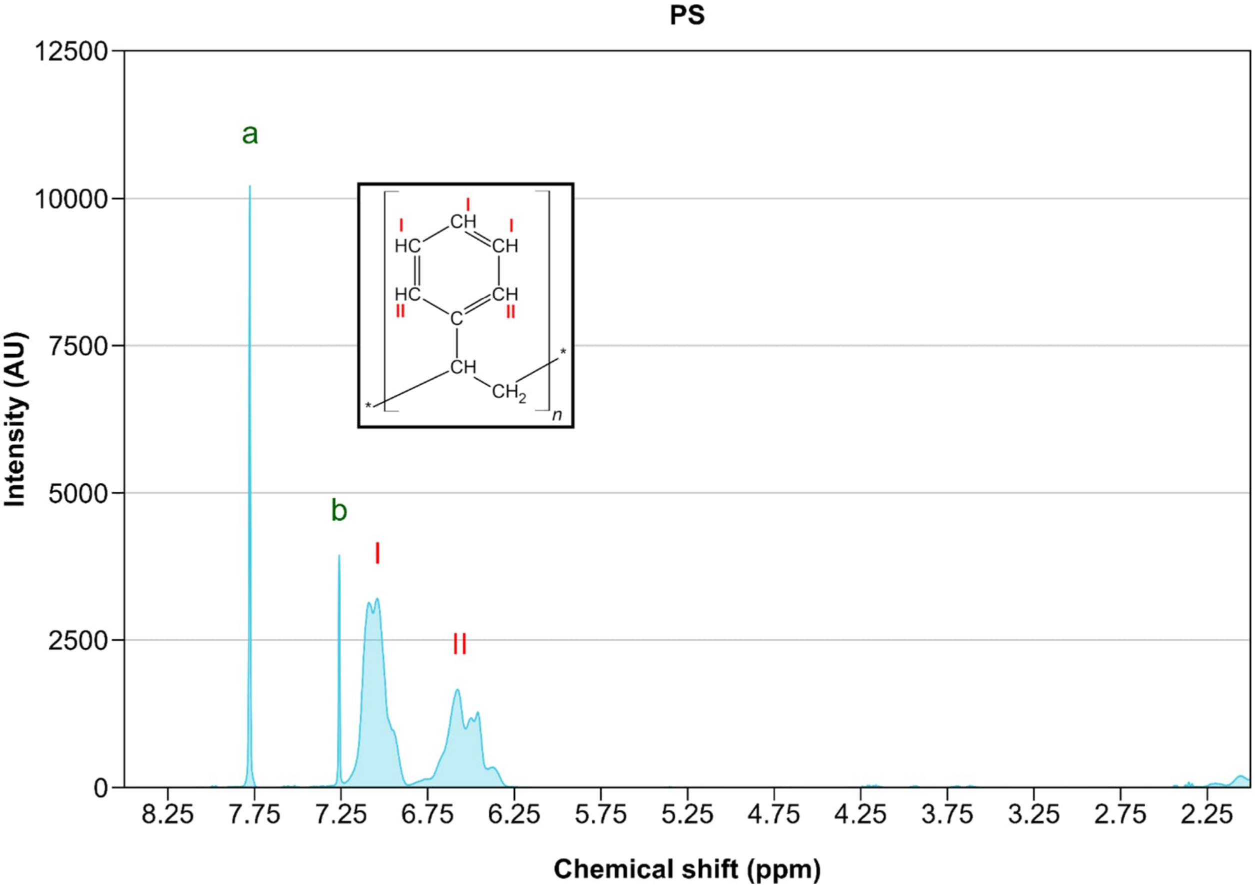

Figure S13. Annotated ^{1}H-NMR spectrum and chemical structure of (atactic) polystyrene (PS). The spectrum was collected in deuterated chloroform. Peak b is the signal from the traces of non-deuterated chloroform (chemical structure also shown in insert). Peak a is the signalfrom the internal standard, 1,4-bis(trifluormethyl)benzene (see Figure S5).

Table S12. Parameters in Eq.1 (manuscript) for the quantification of polystyrene (PS). Peaks are annotated as shown in Figure S13.

Characteristic peak selected for quantification of the repeat unit	I, chemical shift: ~ 6.56 ppm
$\#H_I$	3 protons/repeat unit
Characteristic peak selected for the IS	**a**, chemical shift: ~ 7.78 ppm
$\#H_{IS}$	4 protons/TFB molecule
Resulting form of Eq.1 (manuscript)	$m_{PS} = M_{w_{PS\ unit}} \frac{4}{3} \frac{a_I}{a_{IS}} n_{IS}$
$M_{w_{PS\ unit}}$	104.15 g/mol

^{1}H-NMR spectra of commercial mulch films

The three mulch films used in this study (MF-R, MF-S and MF-E) are composed mainly of PBAT and PLA. Their ^{1}H-NMR spectra are shown in Figure S15 to Figure S17. The parameters in Eq.1 (manuscript) for the quantification of PBAT and PLA are shown in Table S13 and Table S14, respectively.

PBAT is a random copolymer containing two repeat units: the 1,4-butanediol-terephthalic acid (BT) unit and the 1,4-butanediol-adipic acid (BA) unit. As described above for the case of PHBH, the masses of both the BA and BT units need to be quantified separately and added to obtain the total mass of PBAT.

The chemical shift of the α protons in the 1,4-butanediol moieties of PBAT depends on the diacid (terephthalic acid for the BT unit, adipic acid for the BA unit) to which the 1,4-butanediol is bound. However, in the standard chemical structure of PBAT, see Table S3, only one of these (possible) bonds can be shown univocally per each represented diol. To avoid confusion with peak assignments, and particularly with the number of protons contributing to each peak, the following chemical structure (shifted along the polymer chain) was used for annotation in Figure S15 to Figure S17:

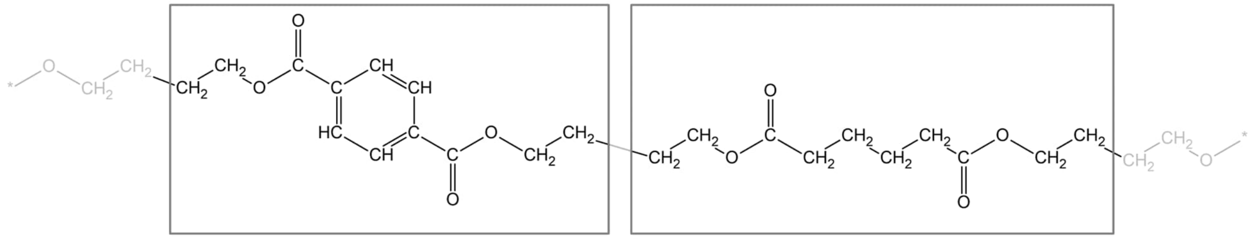

Figure S14. Chemical structure of poly(butylene adipate-co-terephthalate) (PBAT), shifted along the polymer chain to highlight the chemical neighbors of each moiety.

MF-R

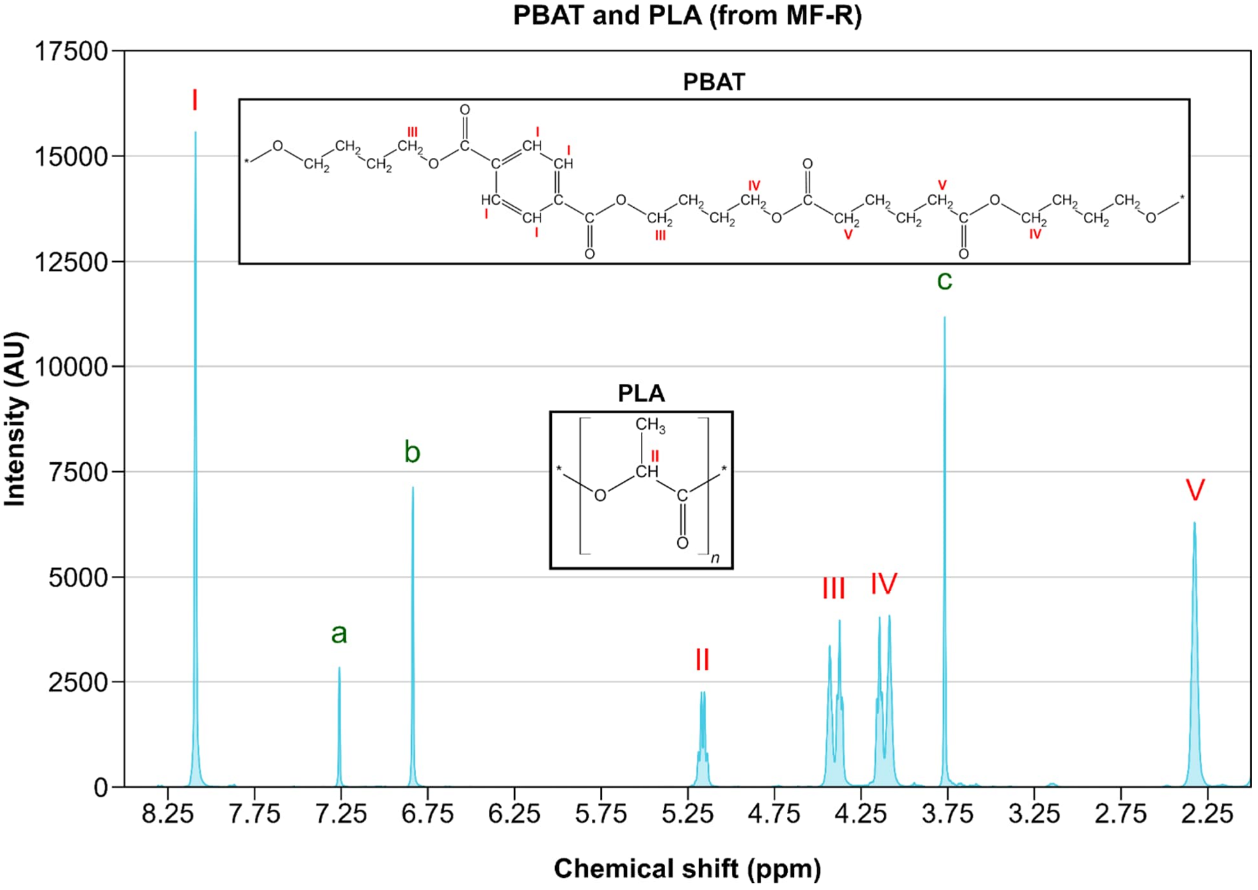

Figure S15. Annotated ^{1}H-NMR spectrum and chemical structures of poly(butylene adipate-co-terephthalate) (PBAT) and polylactic acid (PLA) from the mulch film MF-R (Biofolie '15 µ', Rossat SA, Switzerland). The spectrum was collected in deuterated chloroform. Peak a is the signal from the traces of non-deuterated chloroform in the deuterated solvent and peaks b and c are the signals from the internal standard, 1,4-dimethoxybenzene (see Figure S4).

MF-S

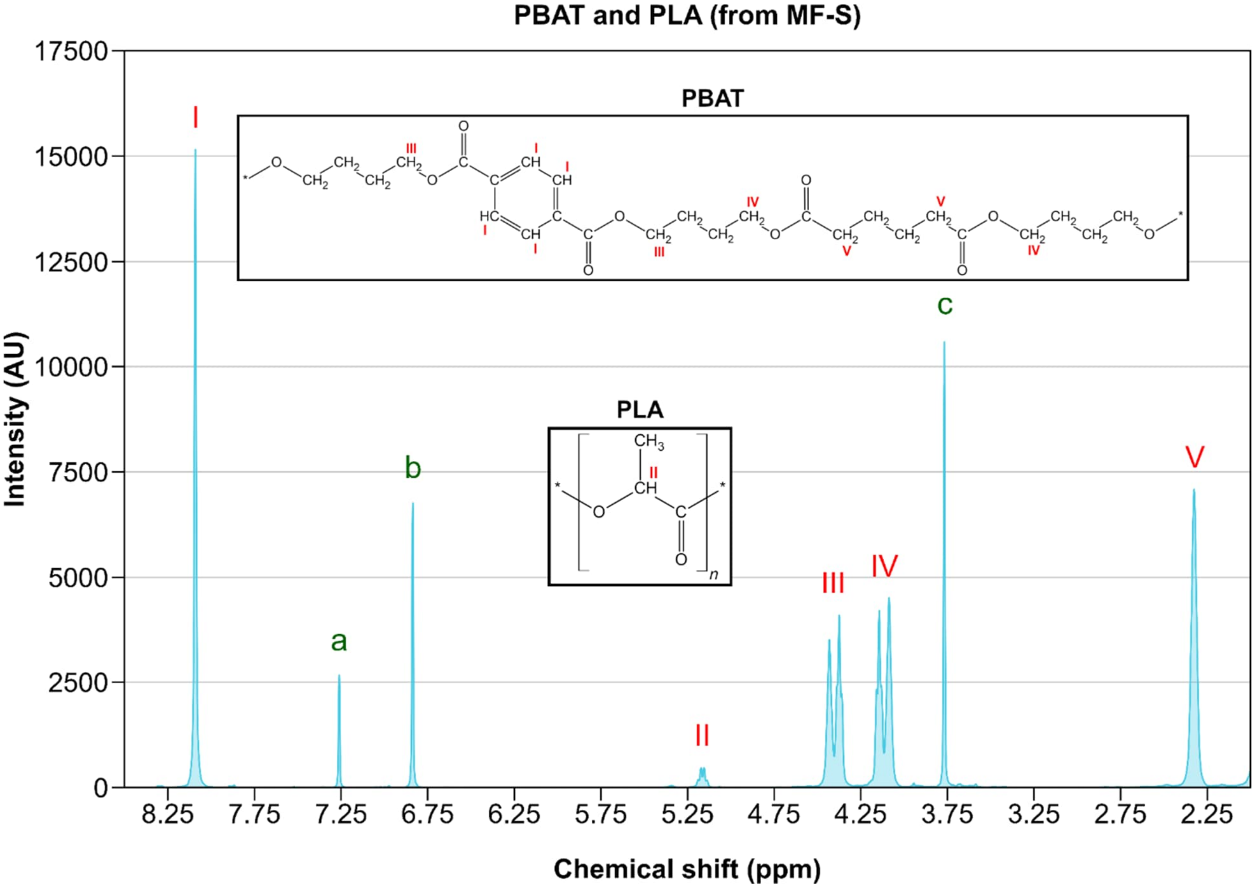

Figure S16. Annotated ^{1}H-NMR spectrum and chemical structures of poly(butylene adipate-co-terephthalate) (PBAT) and polylactic acid (PLA) from the mulch film MF-S (Bio Mulchfolie 32.00009, Sansonnens & Freres SA, Switzerland). The spectrum was collected in deuterated chloroform. Peak a is the signal from the traces of non-deuterated chloroform in the deuterated solvent and peaks b and c are the signals from the internal standard, 1,4-dimethoxybenzene (see Figure S4).

MF-E

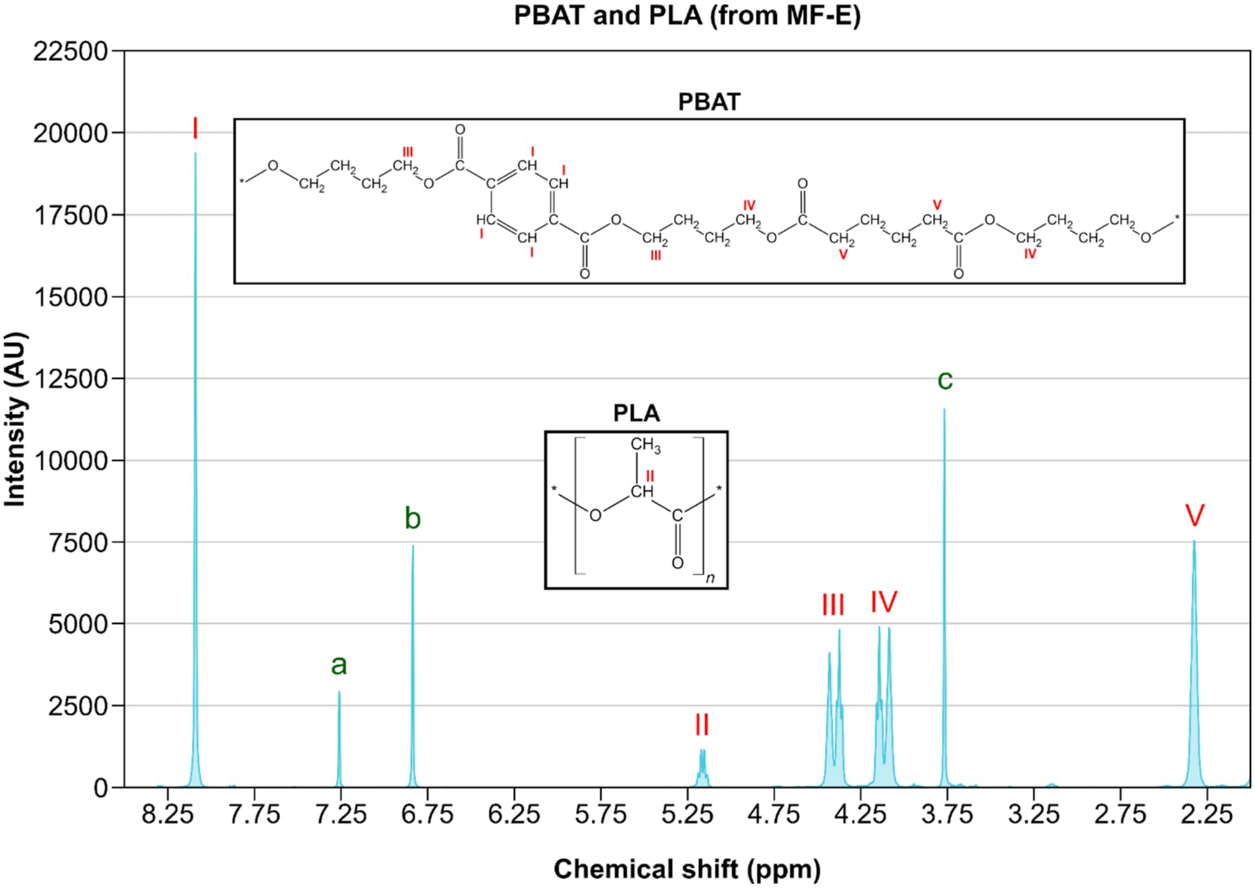

Figure S17. Annotated ^{1}H-NMR spectrum and chemical structures of poly(butylene adipate-co-terephthalate) (PBAT) and polylactic acid (PLA) from the mulch film MF-E (ecovio™ M2351, BASF SE, Germany). The spectrum was collected in deuterated chloroform. Peak a is the signal from the traces of non-deuterated chloroform in the deuterated solvent and peaks b and c are the signals from the internal standard, 1,4-dimethoxybenzene (see Figure S4).

Table S13. Parameters in Eq.1 (manuscript) for the quantification of poly(butylene adipate-co-terephthalate) (PBAT) in the mulch films MF-R, MF-S, and MF-E. Peaks are annotated as shown in Figure S15 to Figure S17.

Characteristic peak selected for quantification of the BT repeat unit	I, chemical shift: ~ 8.09 ppm
$\#H_{I}$	4 protons/repeat unit
Characteristic peak selected for quantification of the BA repeat unit	IV, chemical shift: ~ 4.11 ppm
$\#H_{IV}$	4 protons/repeat unit
Characteristic peak selected for the IS	**b**, chemical shift: ~ 6.84 ppm
$\#H_{IS}$	4 protons/DMB molecule
Resulting form of Eq.1 (manuscript)	$m_{PBAT} = \left(M_{w_{BT}} a_{I} + M_{w_{BA}} a_{IV}\right) \frac{n_{IS}}{a_{IS}}$
$M_{w_{BT\ unit}}$	220.08 g/mol
$M_{w_{BA\ unit}}$	200.06 g/mol

Table S14. Parameters in Eq.1 (manuscript) for the quantification of polylactic acid (PLA) in the mulch films MF-R, MF-S, and MF-E. Peaks are annotated as shown in Figure S15 to Figure S17.

Characteristic peak selected for quantification of the repeat unit	II, chemical shift: ~ 5.17 ppm
$\#H_{II}$	1 proton/repeat unit
Characteristic peak selected for the IS	**b**, chemical shift: ~ 6.84 ppm
$\#H_{IS}$	4 protons/DMB molecule
Resulting form of Eq.1 (manuscript)	$m_{PLA} = M_{w_{PLA\ unit}} 4 \frac{a_{II}}{a_{IS}} n_{IS}$
$M_{w_{PLA\ unit}}$	72.06 g/mol

SECTION 4. R PACKAGES

Table S15. R packages used for data analysis and visualization. Maintainers' information was retrieved on 25th November 2023.

Package	Version	Maintainer	Email
biogeo	V 1.0	Mark P. Robertson	markrobertsonsa@gmail.com
car	V 3.1.3	John Fox	jfox@mcmaster.ca
colorblindcheck	V 1.0.2	Jakub Nowosad	nowosad.jakub@gmail.com
data.table	V 1.14.8	Matt Dowle	mattjdowle@gmail.com
doBy	V 4.6.20	Søren Højsgaard	sorenh@math.aau.dk
extrafont	V 0.19	Winston Chang	winston@stdout.org
geoR	V 1.9.2	Paulo Justiniano Ribeiro Jr	paulojus@ufpr.br
ggh4x	V 0.2.6	Teun van den Brand	tahvdbrand@gmail.com
ggnewscale	V 0.4.9	Elio Campitelli	elio.campitelli@cima.fcen.uba.ar
ggpubr	V 0.6.0	Alboukadel Kassambara	alboukadel.kassambara@gmail.com
ggridges	V 0.5.4	Claus O. Wilke	wilke@austin.utexas.edu
ggsci	V 3.0.0	Nan Xiao	me@nanx.me
ggtern	V 3.4.2	Nicholas Hamilton	nick@ggtern.com
ggthemes	V 4.2.4	Jeffrey B. Arnold	jeffrey.arnold@gmail.com
grateful	V 0.2.4	Francisco Rodriguez-Sanchez	f.rodriguez.sanc@gmail.com
gstat	V 2.1.1	Edzer Pebesma	edzer.pebesma@uni-muenster.de
lattice	V 0.22.5	Deepayan Sarkar	deepayan.sarkar@r-project.org
lme4	V 1.1.35.1	Ben Bolker	bbolker+lme4@gmail.com
metR	V 0.14.1	Elio Campitelli	elio.campitelli@cima.fcen.uba.ar
modeest	V 2.4.0	Paul Poncet	paulponcet@yahoo.fr
nlme	V 3.1.166	R Core Team	R-core@R-project.org
pacman	V 0.5.1	Tyler Rinker	tyler.rinker@gmail.com
pammtools	V 0.5.92	Andreas Bender	andreas.bender@stat.uni-muenchen.de
patchwork	V 1.1.3	Thomas Lin Pedersen	thomasp85@gmail.com
plyr	V 1.8.9	Hadley Wickham	hadley@rstudio.com
pracma	V 2.4.4	Hans W. Borchers	hwborchers@googlemail.com
ragg	V 1.2.6	Thomas Lin Pedersen	thomas.pedersen@posit.co
rcartocolor	V 2.1.1	Jakub Nowosad	nowosad.jakub@gmail.com
Rmisc	V 1.5.1	Ryan M. Hope	rmh3093@gmail.com
sf	V 1.0.14	Edzer Pebesma	edzer.pebesma@uni-muenster.de
sp	V 2.1.1	Edzer Pebesma	edzer.pebesma@uni-muenster.de
tidyverse	V 2.0.0	Hadley Wickham	hadley@posit.co
zoo	V 1.8.12	Achim Zeileis	Achim.Zeileis@R-project.org

SECTION 5. EFFECT OF SOM ON POLYMER QUANTIFICATION

^{1}H-NMR spectra of soil extracts

The ^{1}H-NMR spectra of the soil extracts of soils AGR-2 and LUFA 6S (60 min extraction with 9:1 v/v $CHCl_3$:MeOH, no pre-extraction with MeOH) are shown in Figure S18 and Figure S19.

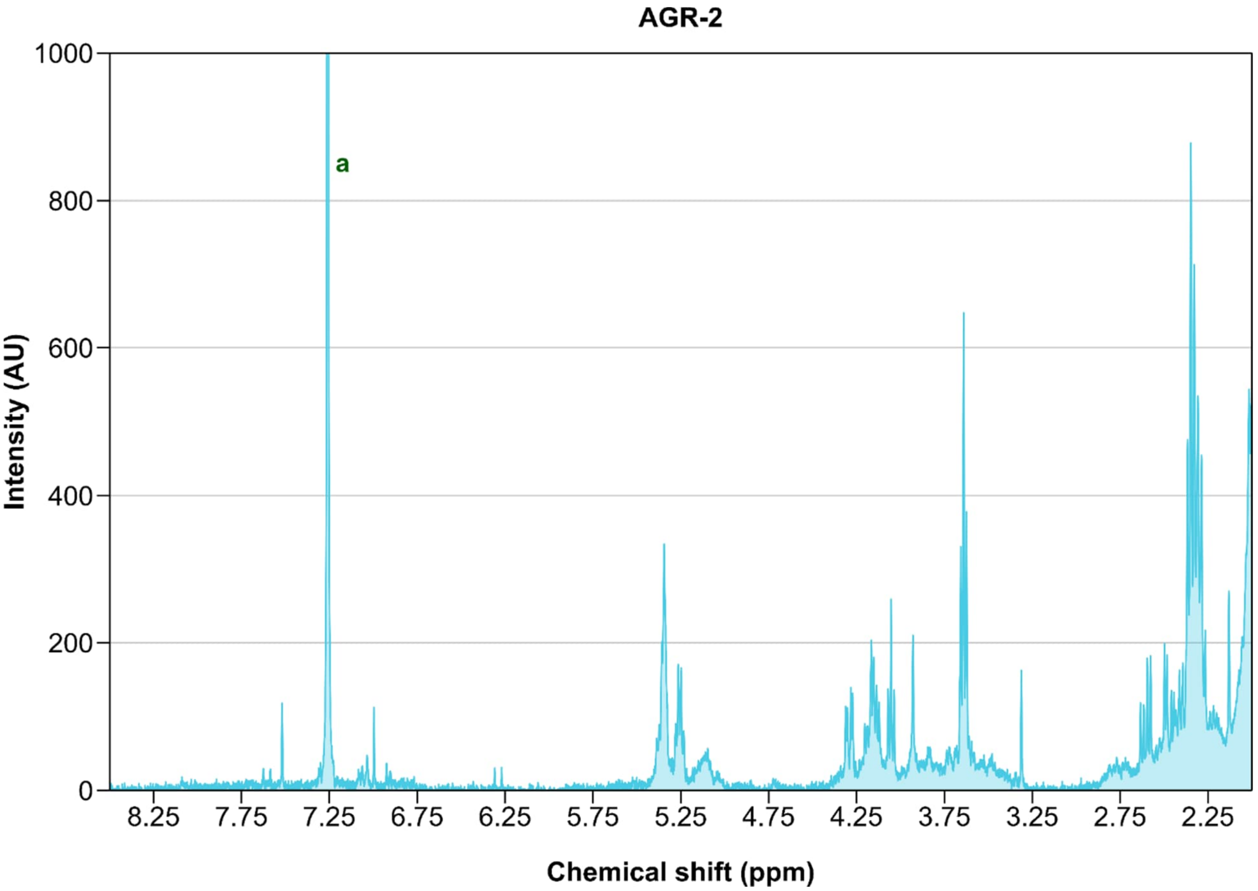

Figure S18. ^{1}H-NMR spectrum of the extract of soil AGR-2 (60 min extraction, 9:1 v/v $CHCl_3$:MeOH). The spectrum was collected in deuterated chloroform. Peak a is the signal from the traces of non-deuterated chloroform in the deuterated solvent.

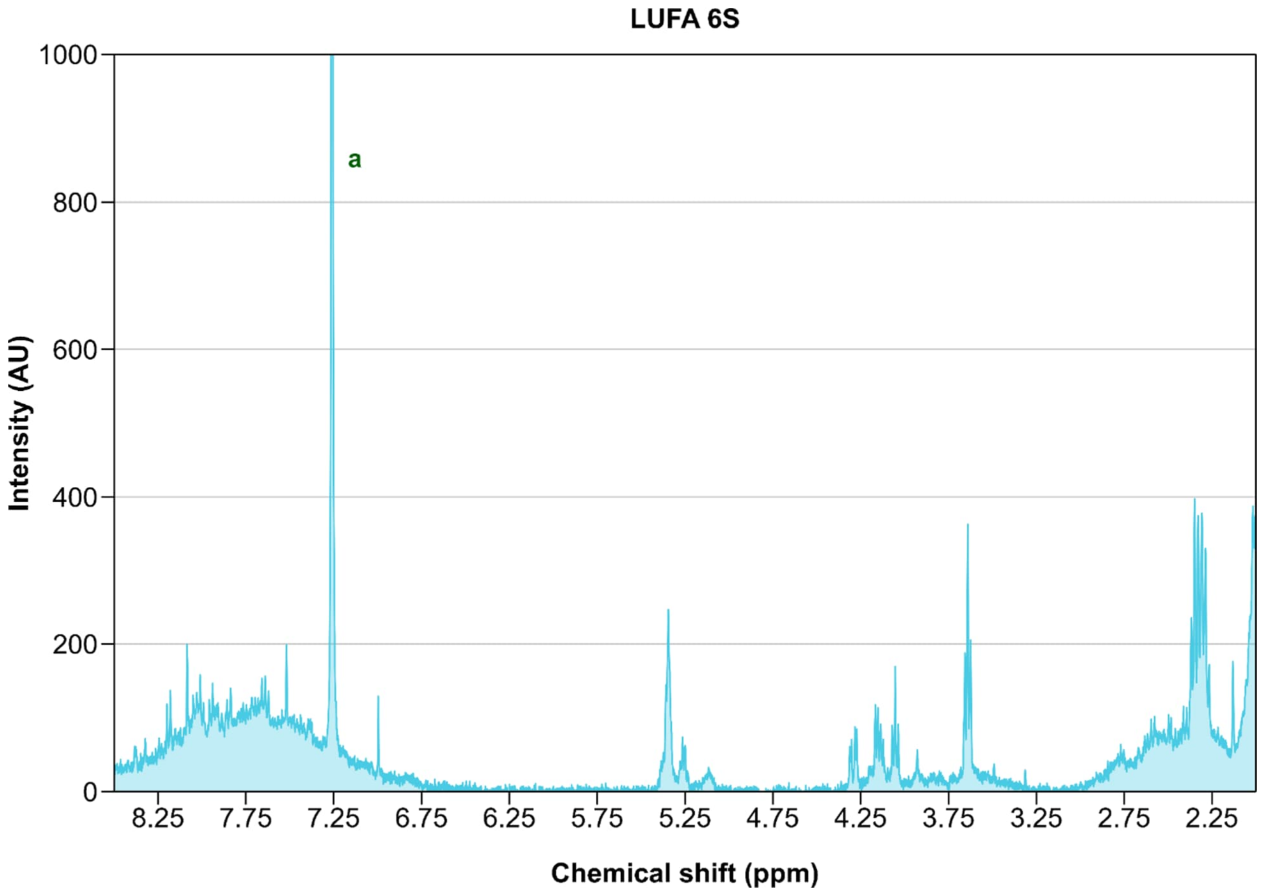

Figure S19. ^{1}H-NMR spectrum of the extract of soil LUFA 6S (60 min extraction, 9:1 v/v $CHCl_3$:MeOH). The spectrum was collected in deuterated chloroform. Peak a is the signal from the traces of non-deuterated chloroform in the deuterated solvent.

Effect of the methanol pre-extraction on co-extraction of SOM

An example of the overlap between signals of the soil extracts from soil AGR-2 and LUFA 6S, with and without MeOH pre-extraction, the soil organic matter that is removed with the MeOH pre-extraction, and the signals of PBAT and PLA from MF-R is shown in Figure S20.

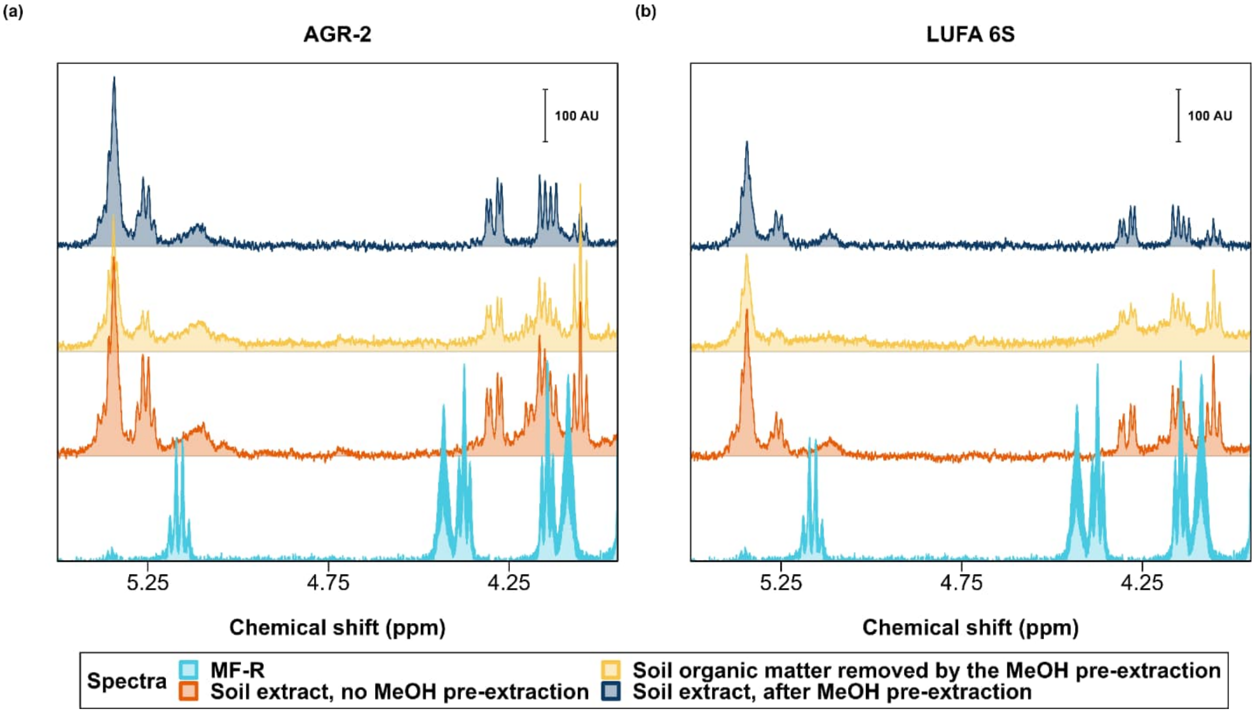

Figure S20. Stacked ^{1}H-NMR spectra of the mulch film MF-R dissolved in pure $CDCl_3$, the extracts of soil AGR-2 and LUFA 6S (60 min extraction, 9:1 v/v $CHCl_3$:MeOH), with and without a MeOH pre-extraction step (30 min pre-extraction, MeOH), and the soil organic matter that is removed with the MeOH pre-extraction step. All extracts (including the MeOH used for the pre-extraction) were dried and reconstituted in $CDCl_3$ for the analysis. The signals correspond to 20 g of soil (either AGR-2 or LUFA 6S) and 17 mg of MF-R.

Calibration curves for the determination of the LOD and LOQ

The limit of detection (LOD) and limit of quantification (LOQ) for the PBAT and PLA components of MF-R were determined for soils AGR-2 and LUFA 6S, using a linear calibration method[7]. The LOD and LOQ were determined for four different situations:

- MF-R dissolved in pure $CDCl_3$ (as a reference),
- MF-R dissolved in the $CHCl_3$:MeOH extract of soils AGR-2 and LUFA 6S (60 min in $CHCl_3$:MeOH, 9:1 v/v),
- MF-R dissolved in the $CHCl_3$:MeOH extract of soils AGR-2 and LUFA 6S (60 min in $CHCl_3$:MeOH, 9:1 v/v) after the soils had already been pre-extracted with MeOH to remove extractable SOM (30 min in MeOH),
- MF-R dissolved in the extract of soil AGR-2 and LUFA 6S (60 min in $CHCl_3$:MeOH, 9:1 v/v) and matrix-matched (i.e., the spectra of the corresponding blank samples containing 0 mg polymer/ml were subtracted from all the others before integration, to correct for the background signals from SOM).

The determination of the LOD and LOQ requires the construction of linear calibration curves (characteristic peak area as a function of the polymer concentration) over an appropriate range of equispaced analyte concentrations[7]: an example of these curves is shown in Figure S21 (LOD of the BA unit of PBAT from MF-R in soil AGR-2 and LUFA 6S).

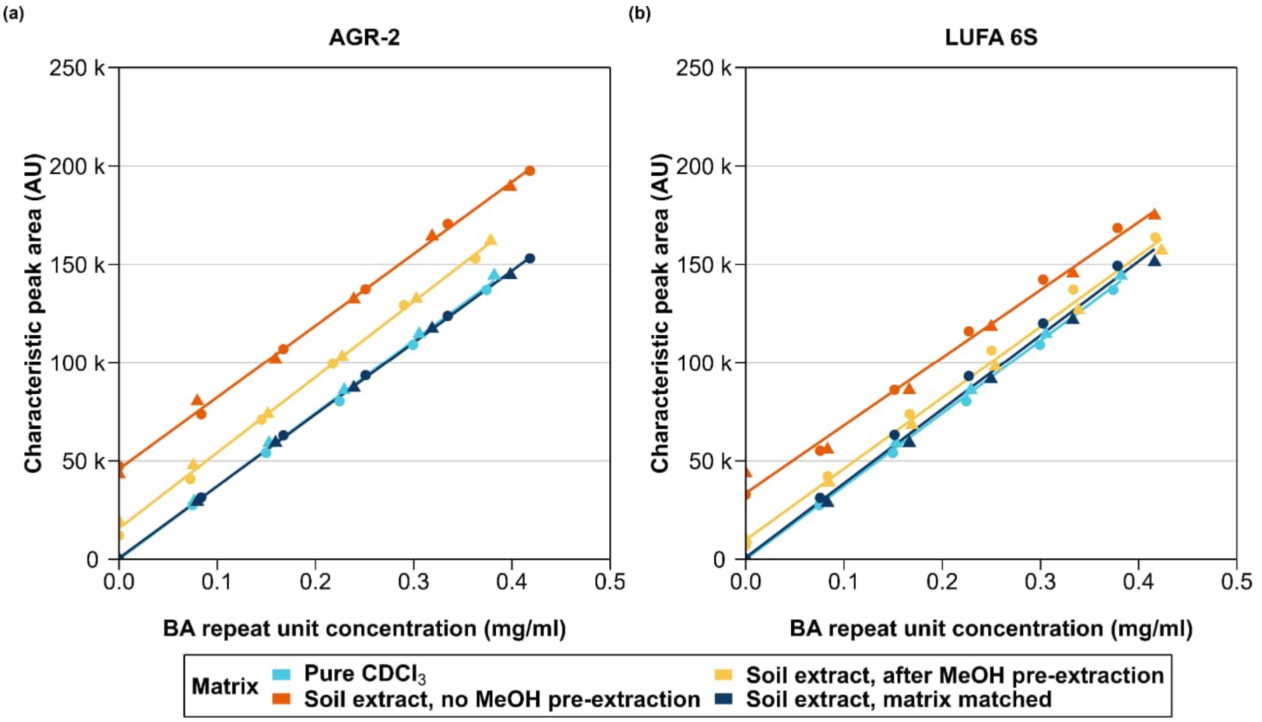

Figure S21. Calibration curves for the determination of the limits of detection and quantification of the 1,4-butanediol-adipic acid repeat unit (BA) of poly(butylene adipate-co-terephthalate) from mulch film MF-R in soils AGR-2 and LUFA 6S. Circles and triangles correspond to duplicates samples.

Effect of the number of measurement scans on the LOD

The intensity of the signals in ^{1}H-NMR spectra is proportional to the number of measurement scans of the NMR routine[8]. If the sample to be analyzed is stable, increasing the number of scans can thus be used to improve the LOD and LOQ. Conversely, if the LOD and LOQ are deemed sufficient, reducing the number of measurement scans can shorten the time required to acquire the ^{1}H-NMR spectra.

A minimal example showing the effect of the number of measurement scans on the LOD is shown in Figure S22. The values shown were generated by measuring the same samples (LOD of the BA unit of PBAT from MF-R in soil AGR-2, with MeOH pre-extraction) with different settings. All other results presented in this study were generated using 128 measurement scans.

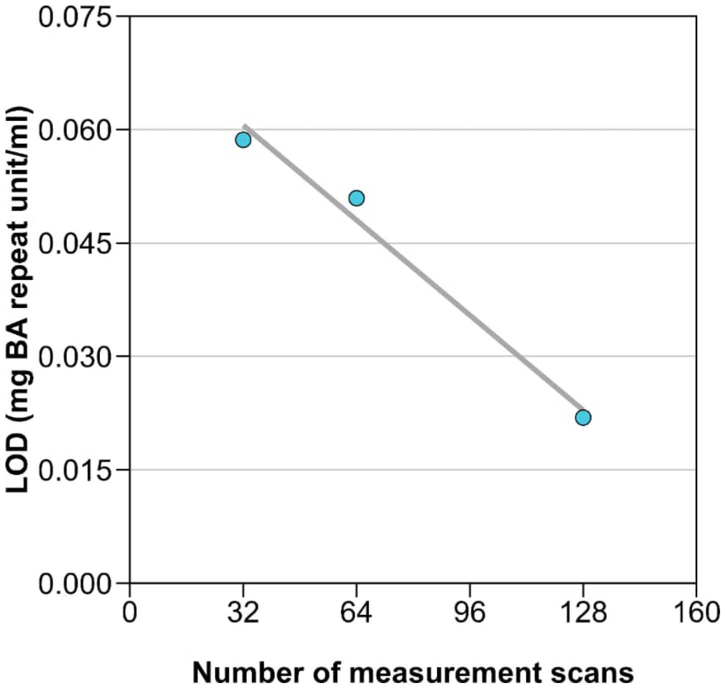

Figure S22. Effect of the number of measurement scans on the limit of detection (LOD) of the 1,4-butanediol-adipic acid repeat unit (BA) of poly(butylene adipate-co-terephthalate) from mulch film MF-R in soil AGR-2 after MeOH pre-extraction.

Comparison of the LOQ to environmental concentrations

There are currently no published data on the (expected) concentration of residual polyesters from biodegradable mulch films in agricultural soils. Therefore, we will compare the LOQ of our analytical method to the expected environmental concentration of residual polyester in an idealized scenario and consider the LOQ of the 1,4-butanediol-adipic acid (BA) repeat unit in PBAT, as an example.

Based on the density of the three mulch films included in the study, a stretch of mulch film of 1 m^2 corresponds to an approximate mass of 18 g film (and approximately 12 g of PBAT or 6 g of the BA repeat unit). To estimate the mass of soil in which the film would be delivered during tillage, let's consider a field section with an area of 1 m^2 and a depth of 20 cm (corresponding to the typical maximum depth during tillage[9]). Assuming a soil density of 1.3 g/cm^3 (typical values range between 1.1 and 1.6 g/cm^3 in most soils[10]), the above described soil volume corresponds to a mass of approximately 260 kg of soil. Finally, it is assumed that ploughing results in a homogeneous distribution of the residual film in the soil, and thus an environmental load of 22 µg BA/g soil.

In all experiments presented in this work, 20 g of soil were extracted into a final solvent volume of 3 ml. If the same sample mass and final solvent volume are maintained for the analysis of the field samples, the expected concentration of the BA repeat unit in the extract would be approximately 146 µg BA/ml. This concentration would be sufficiently high for the quantification of the BA repeat unit in soil AGR-2 (worst case LOQ 86 µg BA/ml, without any additional step to remove the interference from SOM) but not quite in soil LUFA 6S (best case LOQ 152 µg BA/ml, after pre-extraction with MeOH to remove SOM, see also Table 3 in the manuscript).

Realistically, residual mulch film is not homogenously distributed in the soil, but rather incorporated as fragments of various sizes (dm^2 to μm^2, decreasing in size over time). On the top of this, the distribution of these fragments is unlikely to be random (e.g., because films are applied, and ploughed in, in lines and based on crop harvest cycles). This implies that both an appropriate spatial distribution of the samples and the addition of sample volume reduction and homogenization methods[1,11] should be included to accurately estimate environmental concentrations. Our method is, however, sufficiently sensitive to monitor the biodegradation of mulch films using mesh bags, as demonstrated by the mesocosm incubation experiments in the manuscript.

SECTION 6. POLYMER RECOVERIES FOR DIFFERENT EXTRACTION TIMES

The percentage of the total mass of PBAT and PLA (from MF-R) recovered from soil AGR-2 for different total extraction times is shown in Table S16. Note that, although an extraction time of about 10 min would likely be sufficient to achieve complete recovery of these polymers, this was not implemented in any further experiment, as this time was too brief to efficiently operate multiple Soxhlet apparatuses in parallel.

Table S16. Percentage of the total mass of poly(butylene adipate-co-terephthalate) (PBAT) and polylactic acid (PLA) (both from mulch film MF-R) added to soil AGR-2 and extracted for different amounts of time. The extraction time refers to the $CHCl_3$:MeOH extraction of the polymers a(approximately 3 to 4 extraction cycles every 10 minutes). All samples were pre-extracted for 30 min in MeOH to remove extractable soil organic matter beforehand.

Extraction time (min)	Polymer	Extracted polymer (% of added mass, mean ± sd, n=3)
10	PBAT	96 ± 1
	PLA	97 ± 3
30	PBAT	98 ± 1
	PLA	101 ± 2
60	PBAT	100 ± 1
	PLA	102 ± 1
240	PBAT	101 ± 2
	PLA	99 ± 1

SECTION 7. MODEL PROTOCOL

A protocol for the preparation and processing of samples for incubations is included below.

Preparations and sample assembly

There are several steps before the actual sample preparation and burial described below that can be taken care of independently, for example the soil collection and homogenization/sieving (see Section 1), the preparation of soil and polymer aliquots , and the preparation of the mesh bags. Furthermore, an accurate measure of the polymer content of each sample is required to later compare the mass of polymer before and after incubation. Depending on the polymer of interest, this may require additional characterization steps (e.g., in the case of polymer blends, inhomogeneous materials, see Section 2) to ensure accurate polymer quantification.

Figure S23 shows the assembly of a sample: to ensure that the polymer sample (here a disc of mulch film) is surrounded by soil, fill the mesh bag with approximately half of the soil mass (middle image), then place the sample into the mesh bag, and carefully add the remaining soil to fully surround the polymer.

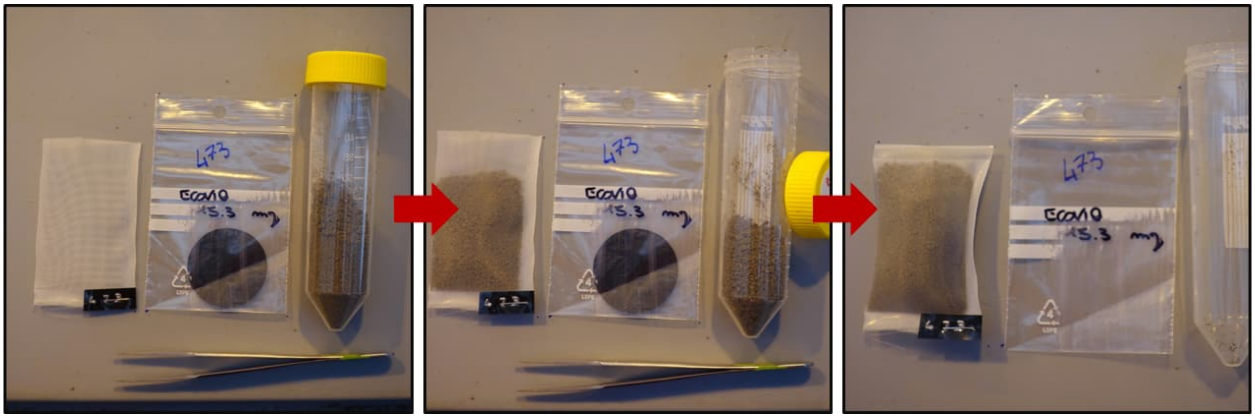

Figure S23. An example of sample assembly with a mulch film disc.

After incubation

Freeze-drying

Retrieve the samples and transfer them to the freezer (-20°C overnight). Freeze-dry the samples to ensure the complete removal of moisture (here: 48 h at 0.01 mbar – note that the duration may need to be adjusted based on the apparatus capacity and sample number).

Grinding

Transfer the dried samples onto glassine (non-stick) paper and remove the soil and polymer from the bag. Manually grind the material using a pestle (note that materials like mulch films may not necessarily have disintegrated into micrometer-sized particles, see Figure S24). Transfer the ground content of the bag and the bag itself to a cellulose extraction thimble and cap the thimble with glass wool to prevent splashing during the extraction. Pay attention not to transfer soil onto the exterior of the thimble.

MeOH pre-extraction step (SOM removal)

For each sample, add 70 ml of MeOH and a Teflon stir bar (to prevent flash boiling) to a 100 ml round bottom flask. Load the extraction thimble in the extractor chamber and connect this to the flask and counter-flow condenser unit. Start the heating apparatus (here: set at 360 W). Once the extraction procedure has been thoroughly tested, it is convenient to extract samples for a fixed amount of time, which then corresponds to a fixed number of extraction cycles. Herein, samples were extracted for 30 min, starting the timer when the extractor chamber drained back into the round bottom flask for the first time.

Once the MeOH extraction has been completed, allow the solvent to drain into the round bottom flask one last time and remove any leftover by manually draining the extractor chamber as well. Disconnect the round bottom flask used for the MeOH extraction.

$CHCl_3$:MeOH extraction step (polymer extraction)

For each sample, add 70 ml of the 9:1 v:v $CHCl_3$:MeOH solvent mix and a Teflon stir bar to a clean 100 ml round bottom flask. Connect the new flask to the extractor chamber and resume the extraction. As for the previous step, it is convenient to extract samples for a fixed amount of time (herein, samples were extracted for 60 min, starting the timer when the extractor chamber drained back into the round bottom flask for the first time). At the end of the extraction, allow the extractor chamber to drain into the round bottom flask one last time, then disconnect the condenser and extractor chamber. Switch off the heating apparatus. Residual heat can be used to evaporate off most of the remaining solvent, but attention must be paid not to char the extracts.

Connect the round bottom flask with the extract to a vacuum line to ensure any leftover solvent is removed (20 min).

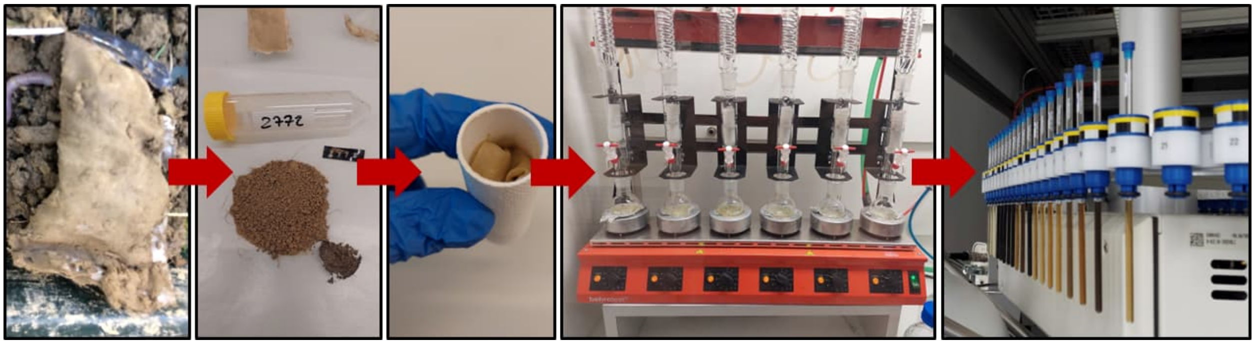

Figure S24. A schematic sequence of the sample processing and extraction steps.

Sample reconstitution

Prepare the reconstitution solvent ($CDCl_3$ containing the desired internal standard in a known concentration). Add 3 ml to each round bottom flask and agitate gently to solubilize the extract and loosen the stir bar from the flask. Sonicate briefly (20 s) to ensure a complete resolubilization. If particulate matter is present in the samples (e.g., pigments that are not chloroform-soluble, or soil accidentally transferred to the round bottom flask during the extraction), it is advisable to

transfer the samples to separate tubes first and centrifuge the particulate out. Transfer the samples (supernatant) to NMR tubes and proceed with the NMR analysis routine.

REFERENCES

(1) Gy, P. Sampling of Discrete Materials — a New Introduction to the Theory of Sampling: I. Qualitative Approach. *Chemometrics and Intelligent Laboratory Systems* **2004**, *74* (1), 7–24. https://doi.org/10.1016/j.chemolab.2004.05.012.

(2) Soil Science Division Staff. *Soil Survey Manual*; USDA Handbook 18; US Government Printing Office: Washington, D.C., 2017. https://www.nrcs.usda.gov/resources/guides-and-instructions/soil-survey-manual (accessed 2025-02-07).

(3) DIN ISO 11277. Soil Quality - Determination of Particle Size Distribution in Mineral Soil Material - Method by Sieving and Sedimentation (DIN ISO 11277:1998 + ISO 11277:1998 Corrigendum 1:2002). *Beuth, Berlin* **2002**.

(4) Hoffmann, G. *VDLUFA-Methodenbuch Band I: Die Untersuchung von Böden*; 1991. https://www.methodenbuch.de/ (accessed 2025-02-07).

(5) Oliver, M. A.; Webster, R. *Basic Steps in Geostatistics: The Variogram and Kriging*; Springer, 2015. https://doi.org/10.1007/978-3-319-15865-5.

(6) Nelson, T. F.; Remke, S. C.; Kohler, H. P. E.; McNeill, K.; Sander, M. Quantification of Synthetic Polyesters from Biodegradable Mulch Films in Soils. *Environmental Science and Technology* **2019**, *54* (1), 266–275. https://doi.org/10.1021/acs.est.9b05863.

(7) European Commission and Joint Research Centre; Robouch, P.; Stroka, J.; Haedrich, J.; Schaechtele, A.; Wenzl, T. *Guidance Document on the Estimation of LOD and LOQ for Measurements in the Field of Contaminants in Feed and Food*; Publications Office, 2016. https://doi.org/10.2787/8931.

(8) Bharti, S. K.; Roy, R. Quantitative 1H-NMR Spectroscopy. *TrAC Trends in Analytical Chemistry* **2012**, *35*, 5–26. https://doi.org/10.1016/j.trac.2012.02.007.

(9) Schneider, F.; Don, A.; Hennings, I.; Schmittmann, O.; Seidel, S. J. The Effect of Deep Tillage on Crop Yield - What Do We Really Know? *Soil and Tillage Research* **2017**, *174*, 193–204. https://doi.org/10.1016/j.still.2017.07.005.

(10) Rai, R. K.; Singh, V. P.; Upadhyay, A. Chapter 17 - Soil Analysis. In *Planning and Evaluation of Irrigation Projects*; Rai, R. K., Singh, V. P., Upadhyay, A., Eds.; Academic Press, 2017; pp 505–523. https://doi.org/10.1016/B978-0-12-811748-4.00017-0.

(11) Gy, P. Sampling of Discrete Materials: II. Quantitative Approach—Sampling of Zero-Dimensional Objects. *Chemometrics and Intelligent Laboratory Systems* **2004**, *74* (1), 25–38. https://doi.org/10.1016/j.chemolab.2004.05.015.
